# How well do critical care audit and feedback interventions adhere to best practice? Development and application of the REFLECT-52 evaluation tool

**DOI:** 10.1186/s13012-021-01145-9

**Published:** 2021-08-17

**Authors:** Madison Foster, Justin Presseau, Eyal Podolsky, Lauralyn McIntyre, Maria Papoulias, Jamie C. Brehaut

**Affiliations:** 1 School of Epidemiology and Public Health, University of Ottawa, 451 Smyth Road, Ottawa, ON K1H 8M5 Canada; 2 Ottawa Hospital Research Institute, Clinical Epidemiology Program, The Ottawa Hospital, General Campus, 501 Smyth Road, Centre for Practice Changing Research, Box 201B, Ottawa, ON K1H 8L6 Canada; 3 School of Psychology, University of Ottawa, 136 Jean-Jacques Lussier, Vanier Hall, Ottawa, ON K1N 6N5 Canada; 4 Department of Critical Care Medicine, The Ottawa Hospital, General Campus, 501 Smyth Road, Ottawa, ON K1H 8L6 Canada

**Keywords:** Audit, Feedback, A&F, Evaluation tool, Critical care

## Abstract

**Background:**

Healthcare Audit and Feedback (A&F) interventions have been shown to be an effective means of changing healthcare professional behavior, but work is required to optimize them, as evidence suggests that A&F interventions are not improving over time. Recent published guidance has suggested an initial set of best practices that may help to increase intervention effectiveness, which focus on the “Nature of the desired action,” “Nature of the data available for feedback,” “Feedback display,” and “Delivering the feedback intervention.” We aimed to develop a generalizable evaluation tool that can be used to assess whether A&F interventions conform to these suggestions for best practice and conducted initial testing of the tool through application to a sample of critical care A&F interventions.

**Methods:**

We used a consensus-based approach to develop an evaluation tool from published guidance and subsequently applied the tool to conduct a secondary analysis of A&F interventions. To start, the 15 suggestions for improved feedback interventions published by Brehaut et al. were deconstructed into rateable items. Items were developed through iterative consensus meetings among researchers. These items were then piloted on 12 A&F studies (two reviewers met for consensus each time after independently applying the tool to four A&F intervention studies). After each consensus meeting, items were modified to improve clarity and specificity, and to help increase the reliability between coders. We then assessed the conformity to best practices of 17 critical care A&F interventions, sourced from a systematic review of A&F interventions on provider ordering of laboratory tests and transfusions in the critical care setting. Data for each criteria item was extracted by one coder and confirmed by a second; results were then aggregated and presented graphically or in a table and described narratively.

**Results:**

In total, 52 criteria items were developed (38 ratable items and 14 descriptive items). Eight studies targeted lab test ordering behaviors, and 10 studies targeted blood transfusion ordering. Items focused on specifying the “Nature of the Desired Action” were adhered to most commonly—feedback was often presented in the context of an external priority (13/17), showed or described a discrepancy in performance (14/17), and in all cases it was reasonable for the recipients to be responsible for the change in behavior (17/17). Items focused on the “Nature of the Data Available for Feedback” were adhered to less often—only some interventions provided individual (5/17) or patient-level data (5/17), and few included aspirational comparators (2/17), or justifications for specificity of feedback (4/17), choice of comparator (0/9) or the interval between reports (3/13). Items focused on the “Nature of the Feedback Display” were reported poorly—just under half of interventions reported providing feedback in more than one way (8/17) and interventions rarely included pilot-testing of the feedback (1/17 unclear) or presentation of a visual display and summary message in close proximity of each other (1/13). Items focused on “Delivering the Feedback Intervention” were also poorly reported—feedback rarely reported use of barrier/enabler assessments (0/17), involved target members in the development of the feedback (0/17), or involved explicit design to be received and discussed in a social context (3/17); however, most interventions clearly indicated who was providing the feedback (11/17), involved a facilitator (8/12) or involved engaging in self-assessment around the target behavior prior to receipt of feedback (12/17).

**Conclusions:**

Many of the theory-informed best practice items were not consistently applied in critical care and can suggest clear ways to improve interventions. Standardized reporting of detailed intervention descriptions and feedback templates may also help to further advance research in this field. The 52-item tool can serve as a basis for reliably assessing concordance with best practice guidance in existing A&F interventions trialed in other healthcare settings, and could be used to inform future A&F intervention development.

**Trial registration:**

Not applicable.

**Supplementary Information:**

The online version contains supplementary material available at 10.1186/s13012-021-01145-9.

Contributions to the literature

- A recent publication provided several suggestions for best practice in the development, design, and delivery of Audit and Feedback (A&F) interventions; however, the extent of their use in existing interventions is unclear.
- We developed a tool to enable assessment of concordance of A&F interventions with best practice recommendations and tested the tool using a sample of A&F interventions delivered in critical care to improve test and transfusion ordering.
- There is considerable room for improving in the reporting and application of these suggestions for best practice in critical care.
- This tool can be used as a guide for evaluating and designing A&F interventions that adhere to current best practice guidance.

## Background

Audit and feedback (A&F) (i.e., summarizing provider behavior and feeding the data back to them as a means to spur practice change) is a popular class of healthcare professional behavior change interventions [1]. Despite clear evidence that A&F is generally effective in improving care, effect sizes across trials of A&F interventions range from relatively large (25% of studies showed a 16% improvement or better) to null or even negative effects [1]. This variation has important implications. In some cases, it is possible for A&F to reduce the quality of care; if A&F is not optimally delivered, providers’ performance (and the care received by patients) may be negatively impacted, and resources wasted. Finding ways to optimize A&F in healthcare is a clear priority [1–3].

Recent guidance summarized suggestions for optimizing A&F, compiling lessons from interviews with experts in A&F theory and practical team experience, to produce 15 theory-informed suggestions for high quality A&F interventions (Table 1) [4]. These suggestions focus on easily modifiable elements of A&F proposed to improve effectiveness of these interventions to improve behavior change, including the “Nature of the desired action,” “Nature of the data available for feedback,” “Feedback display,” and “Delivering the feedback intervention.” While these suggestions appear to be helping in the development of new A&F interventions [5], they are broadly described and the extent to which published A&F intervention studies already adhere to them remains unclear. A tool to enable detailed assessment of concordance with these suggestions is needed to enable evidence to accrue on which aspects of A&F best practice are being used and which could be optimized in a given literature and setting.

**Table 1:** Evaluation tool criteria items organized by Brehaut and colleagues’ 15 suggestions for improved audit and feedback interventions [4]

15 Suggestions	Criteria Items	Response Scale
**Nature of the desired action**		
1. Recommend actions that are consistent with established goals and priorities	1.1 Is there any indication that the recipients set an internal goal for themselves (i.e. a specific, numerical target/threshold)?	Yes/ No/ Not Reported/ Unclear
	1.2 Was feedback presented in the context of an external, explicit priority?	Yes/ No/ Not Reported/ Unclear
	1.3 If yes, at what level was the priority set?	National/Federal, Provincial/State, Municipal, Institutional, Departmental, Individual-healthcare providers, Individual- researchers, Other (Please specify), Not Reported, Unclear, N/A
	1.4 Does the feedback directly address one or more of the goals or priorities?	Yes/ No/ Not Reported/ Unclear/ N/A
2. Recommend actions that can improve and are under the recipient’s control	2.1 Was feedback on performance provided to allow current performance to be compared against previous performance?	Yes/ No/ Not Reported/ Unclear
	2.2 Does the feedback (or the description of the feedback) describe or show a discrepancy between recipient performance and the goal/ benchmark/ target/comparator?	Yes/ No/ Not Reported/ Unclear
	2.3 Is it reasonable that the feedback recipient can be responsible for the change in behavior?	Yes/ No/ Not Reported/ Unclear
	2.4 Does the feedback provide data on behaviors, outcomes, or both?	For each, answer: Yes/ No/ Not Reported/ Unclear
3. Recommend specific actions	3.1 Did the feedback intervention incorporate suggested corrective actions to support plans for problem solving? For example: action plans, coping strategies, a menu of options, etc.	Yes/ No/ Not Reported/ Unclear
**Nature of the data available for feedback**		
4. Provide multiple instances of feedback	4.1 Was feedback (for a given behavior) provided more than once?	Yes/ No/ Not Reported/ Unclear/ Other
	4.2 Did recipients continue to receive feedback on their performance after the study was completed?	Yes/ No/ Not Reported/ Unclear
5. Provide feedback as soon as possible and at a frequency informed by the number of new patient cases	5.1 What is the average age of the data (i.e. interval between the clinical encounter and delivery of the feedback)?	Describe (i.e. Days, Weeks, Months, Years, Not Reported, Unclear)
	5.2 If feedback was provided more than once, what was the time interval between the receipt of feedback reports?	Describe (i.e. Hours, Days, Weeks, Months, Years, Variable, Not Reported, Unclear, N/A)
	5.3 Do the authors of the study provide a justification for the interval between feedback reports?	Yes/ No/ Unclear/ N/A
	5.4 Was the justification for the interval between feedback reports related to the number of patient cases?	Yes/ No/ Unclear/ N/A
6. Provide individual rather than general data	6.1 Was feedback given about the individual’s own performance?	Yes/ No/ Not Reported/ Unclear
	6.2 Was feedback about the performance of a group of which the recipient is a member?	Yes/ No/ Not Reported/ Unclear
	6.3 What is the level of the group?	Describe: Unit, Department, Practice, Hospital, Region, Province, Other (please specify), Not Reported, N/A
	6.4 Did feedback include patient- level data for the recipient’s own patients?	Yes/ No/ Not Reported/ Unclear
	6.5 Did feedback include aggregated patient data involving recipient’s own patients?	Yes/ No/ Not Reported/ Unclear
	6.6 Do the authors give a justification for the specificity of the feedback (or a reason for the level of data presented)?	Yes/ No/ Unclear
7. Choose comparators that reinforce desired behavior change	7.1 Did the feedback provide any comparators?	Yes/ No/ Not Reported/ Unclear
	7.2 How many comparators were provided?	1, 2, 3, More than 3, None, Not Reported, Unclear, Other (describe as needed)
	7.3 Describe all that apply (e.g. Own previous performance, Other’s performance, Benchmark/ Standardized Guideline/Target, Other, Not Reported, Unclear)	Own previous performance, Other's performance, Benchmark/ Standardized Guideline/Target, Other, Not Reported, Unclear
	7.4 Does feedback include one or more aspirational comparators (as opposed to average performance comparators)?	Yes/ No/ Not Reported/ Unclear/ Other
	7.5 Do the authors provide a justification for which comparators were used?	Yes/ No/ Unclear/ N/A
**Feedback display**		
8. Closely link the visual display and summary message	8.1 Are the visual display and the summary message presented in visual proximity of each other?	Yes/ No/ Not Reported/ Unclear/ N/A
9. Provide feedback in more than one way	9.1 Was feedback provided in more than one way?	Yes/ No/ Not Reported/ Unclear
	9.2 Does the feedback intervention include: 1) Verbal interaction; 2) Text; 3) Numerical information; 4) Graphs or tables; 5) A summary message; 6) Other important elements (please specify)?	Provide an answer for each item: Yes/ No/ Not Reported/ Unclear
10. Minimize extraneous cognitive load for feedback recipients	10.1 Was the feedback intervention pilot-tested?	Yes (with target population)/ Yes (with non-target population)/ No/ Not Reported, Unclear
	10.2 How long is the feedback report?	Describe
	10.3 How many behaviors does the feedback address?	1, 2, 3, 4, 5–9, more than 9, Not Reported, Unclear, Other (describe as needed), N/A
	10.4 How many clinical variables were fed back to the recipients?	Describe/ Not Reported/ Unclear
	10.5 How many graphs or tables are used?	Describe/ Not Reported/ Unclear
	10.6 Did the feedback include any graphical elements that lend themselves to misinterpretation? (Ex. Pie charts, 3D graphs, etc.)	Yes/ No/ Not Reported/ Unclear/ N/A
**Delivering the feedback intervention**		
11. Address barriers to feedback use	11.1 Were potential drivers and barriers to recipients engaging with the feedback component of the intervention assessed?	Yes/ No/ Not Reported/ Unclear
	11.2 Was the assessment informed by theory?	Yes/ No/ Not Reported/ Unclear/ N/A
	11.3 Was there an assessment of whether the recipients engaged with the feedback?	Yes/ No/ Not Reported/ Unclear/ Other
	11.4 Was the assessment informed by theory?	Yes/ No/ Not Reported/ Unclear/ N/A
12. Provide short, actionable messages followed by optional detail	12.1 Are the summary messages actionable (or described as actionable)?	Yes/ No/ Not Reported/ Unclear/ N/A
	12.2 Is there additional, more detailed feedback provided alongside the summary message?	Yes/ No/ Not Reported/ Unclear
13. Address credibility of the information	13.1 Does the feedback (or the description of the feedback) indicate who is providing the feedback data?	Yes/ No/ Not Reported/ Unclear
	13.2 Does the feedback intervention indicate the source of comparators?	Yes/ No/ Not Reported/ Unclear/ Other
	13.3 Was the feedback intervention delivered by a supervisor or close colleague?	Yes/ No/ Not Reported/ Unclear
	13.4 Was the feedback intervention supported by a relevant professional organization?	Yes/ No/ Not Reported/ Unclear
14. Prevent defensive reactions to feedback	14.1 Did the feedback intervention include reassurance that the intervention would not trigger punitive measures?	Yes/ No/ Not Reported/ Unclear/ Other
15. Construct feedback through social interaction	15.1 Did development of the feedback intervention involve members of the target group?	Yes/ No/ Not Reported/ Unclear
	15.2 Was the feedback explicitly designed to be received and discussed in a social context?	Yes/ No/ Not Reported/ Unclear/ Other
	15.3 If feedback was provided more than once, how often was feedback received and discussed in a social context?	Every time feedback was provided, Only once, Variably, Not Reported, Unclear, Other (please specify), N/A
	15.4 If feedback was received and discussed in a social context, was the feedback discussion facilitated by a facilitator?	Yes/ No/ Not Reported/ Unclear/ N/A
	15.5 Did the feedback intervention involve engaging in self- assessment around target behaviors prior to receiving feedback?	Yes/ No/ Not Reported/ Unclear
	15.6 Did the investigators actively seek feedback from the recipients on the feedback?	Yes/ No/ Not Reported/ Unclear

A&F may be a particularly well-suited intervention to change behavior in complex environments such as critical care. In this setting, critically ill patients are rigorously monitored in intensive care units (ICUs) and treated by interdisciplinary teams of healthcare providers, composed of individuals from various professional backgrounds [6–8]. Due to the severity of patient illness, the ICU is a fast-paced and high pressure environment [9, 10]. This creates a stressful workplace, not only emotionally (due to the requirement to make difficult decisions quickly), but also as a result of physical and professional factors [10]. Poor lighting, alarms with low sensitivity, and similar sounds for different warnings, poorly placed equipment, and a multitude of cords and tubes have been cited as physical factors adding to the stressful environment of the ICU [6, 10]. ICUs also produce a large amount of patient data (i.e., vital signs and laboratory data), which can be difficult for individual providers to process [6, 11].

Many behaviors within critical care (test ordering, transfusion ordering) can become routine [12] such that those ordering may not be as aware of the frequency with which orders are being made. This may in turn lead to potentially unnecessary blood draws, putting patients at risk for anemia, and increased cost of care and resources to collect, run, and interpret tests [13–18], or potentially unnecessary use of precious blood products [18–25]. Providing performance data on routinized behaviors may help to highlight the frequency with which these orders are placed and flag them for improvement. Feedback can be provided to both individuals and groups in a variety of ways, which may be useful in addressing the team-based and multidisciplinary [7, 10] nature of the critical care setting. Moreover, data on common practices like laboratory test ordering in this setting are readily available to allow for auditing and production of feedback reports.

We recently conducted a systematic review [26] of the use and effectiveness of A&F interventions in critical care. In the current study, we sought to assess the extent to which these identified A&F intervention studies included Brehaut et al.’s 15 suggestions [4]. These suggestions were designed to provide general guidance to feedback developers (those who actively design A&F displays, e.g., information technology developers, researchers, quality improvement professionals) with each suggestion encompassing multiple concepts that can be applied in a variety of different ways. To better assess how existing A&F interventions may adhere to these suggestions, we aimed to develop an evaluation tool by deconstructing each of the 15 suggestions into unidimensional items that could be reliably rated. Our objectives for this study were to report the development of this evaluation tool, as well as an initial testing of this tool through application to a sample of published A&F intervention studies in the field of critical care.

## Methods

A consensus-based approach was used to develop the evaluation tool from Brehaut et al.’s suggestions for improved A&F. A secondary analysis of A&F interventions was also undertaken to apply the tool and assess the extent to which these practices are observed in the critical care literature. Given the descriptive nature of the evaluation tool, and our narrative approach to reporting the development and application of the tool, results were reported as per the consolidated criteria for reporting qualitative studies (COREQ) checklist (Additional File 1) [27].

### Development of items and response categories

Items were developed by the research team (JCB, JP, MF, MP). First, suggestions encompassing more than one distinct concept were split into items that addressed a single concept. Next, items were worded to facilitate reliable rating of A&F interventions using an iterative process, whereby items were discussed until consensus was reached that the items adequately represented the key components of each suggestion. Items were then de-duplicated, and any items that were judged likely to be difficult to assess from published articles or feedback templates were removed.

Response categories, item-specific anchors, and examples of adherence to increase inter-rater reliability were also developed as part of the coding manual. The response categories (Yes/No/Unclear/Not Applicable) were chosen for most items to facilitate quantitative summaries (“ratable” items, wherein adherence could be determined); the remaining “descriptive” items used a combination of descriptive or numerical response categories, to provide further details about the A&F intervention component (e.g., number of comparators, type of comparators).

### Evaluation tool piloting

A sample of A&F intervention studies were selected from the 2012 A&F Cochrane Review [1] (from outside of the critical care setting, and not necessarily focused on test or transfusion ordering) to pilot the evaluation criteria and assess inter-rater reliability. Each sample, containing four A&F interventions, was independently rated with the pilot criteria by two raters (MF and MP). Consensus meetings were held after application to each sample, to compare data extraction results between raters, discuss discrepancies, and modify the wording of items as necessary to improve their clarity, rateability, and mutual exclusivity. The descriptive anchors and examples were also updated and added to as needed. Disagreements between raters were resolved by a third individual (JCB). Twelve different A&F intervention studies were rated in total. Inter-rater reliability was measured by tabulating agreement scores and calculating Cohen’s Kappa in Microsoft Excel, for the ratable items (items which used “Yes/No/Unclear/Not Applicable” response categories) (Additional File 2) [28]. The pilot study concluded once all ambiguities had been clarified and the research team agreed that the criteria items comprehensively covered all 15 suggestions [4].

### Identification and collection of study materials for application of the evaluation tool

Studies evaluating A&F interventions that were targeted to improve laboratory test and transfusion (red blood cell, platelet, plasma, cryoprecipitate) ordering in a critical care setting were previously identified through a systematic review [26]. The review summarized the current evidence on the use of A&F for quality improvement of lab test and transfusion ordering decisions in critical care. Sixteen studies (17 publications) were identified; six of which aimed to improve lab test ordering [29–35], eight of which aimed to improve blood transfusion ordering [36–43], and two of which assessed both types of orders [44, 45]. Corresponding authors from all 17 publications were contacted by email to request a template of the feedback form used and any other pertinent details about the intervention.

### Application of the evaluation tool

#### Data extraction

After development and pilot testing, we used the evaluation tool to assess the sample of 17 A&F interventions (from 16 studies) [29–45] identified by our previous systematic review [26]. One reviewer (MF) extracted data both from published reports and, when provided by authors, the sample feedback forms. Data was collected using a data extraction form in Microsoft Excel; data extraction was then confirmed by a second reviewer (EP). Disagreements were resolved through consensus, or when an agreement could not be reached, through input from a third reviewer (JCB).

#### Analysis

Descriptive statistics for the criteria items (the number of A&F interventions coded to each response category) were computed and tabulated manually in Microsoft Excel. Results are described in the text and presented graphically (rateable items) or in a table (descriptive items). Gaps in the current literature (items with low adherence) were also identified and discussed narratively.

### Ethics

This study was approved by the Ottawa Health Science Network Research Ethics Board (OHSN-REB; Protocol ID: 20160951-01H).

## Results

### Development and piloting of the evaluation tool

Through iterative team discussions, the 15 suggestions were deconstructed into 39 ratable items and 12 descriptive items. After the pilot, a Cohen’s Kappa of 0.58 was computed for the ratable items. This Kappa score represents “moderate agreement” as per Landis and Koch, but is below Krippendorff’s cut-off “suggesting that conclusions should be discounted” [28]. This relatively low agreement score was partially driven by discrepancies in determining whether the item was not present (“no”) versus “not applicable” or “unclear.” We therefore proceeded to use our primary approach of establishing consensus for each study in the final assessment. Through the pilot consensus meetings, it was determined that two ratable criteria items should be removed (deemed redundant or too difficult to assess), two new items were developed (one ratable and one descriptive), and one descriptive item was re-worded to a ratable item. This resulted in a total of 39 ratable and 12 descriptive items for the application of the evaluation tool. The response scale for the descriptive item *“Does the feedback intervention include: (a) verbal interaction, (b) text, (c) numerical information, (d) graphs or tables, (e) a summary message, (f) other important elements”* was also adjusted such that reviewers were to answer “Yes/No/Unclear/Not Applicable” for each sub-category. “Not reported” was also later added as a response category for all items in the final assessment to differentiate between cases where the answer could not be determined due to lack of reporting or lack of access to the feedback form, as compared to cases where the answer was a clear “No” or “Unclear” (e.g., ambiguous wording or statement).

Post hoc, it was determined that two ratable items (“*Does the feedback include group performance data for which the recipient is a member*?” and “*Does feedback include aggregated patient data involving the recipient’s own patients*?”) were better suited as descriptive items, as adherence to the overall suggestion could not be directly determined. An additional ratable item (“*Was feedback provided in more than one way*?”) was also added ad hoc, to summarize findings from the descriptive item “*Does the feedback intervention include*: (*a*) *verbal interaction*, (*b*) *text*, (*c*) *numerical information*, (*d*) *graphs or tables*, (*e*) *a summary message*, (*f*) *other important elements*.” In the final application of the tool, there were thus 38 ratable items and 14 descriptive items.

Table 1 describes the final 52 criteria items deconstructed and operationalized from the 15 suggestions (Nature of the Desired Action = 9 items; Nature of the Data Available for Feedback = 17 items; Feedback Display = 9 items; Delivering the Feedback Intervention = 17 items). The full evaluation tool, including anchors, is described in Additional File 3.

### Identification of studies and collection of study materials for application of the evaluation tool

Sixteen studies (17 publications) describing 17 A&F interventions (one study compared two different types of feedback) were identified by our systematic review [26]. Additional File 4 provides a flow diagram of the study selection. One study presented an example of the feedback form within the publication, while another presented a portion of the feedback form (the feedback data graph) within the publication. Four authors were able to provide an example of the feedback form utilized in the study as well as additional pertinent details (e.g., whether verbal feedback was provided), and two authors responded but were unable to provide further details (response rate = 6/17 articles (35%). As one of the authors provided examples for the study with two types of A&F interventions, we received feedback forms for five of the 17 interventions (29%); including the two examples provided within the publications, we had access to forms for seven of the 17 interventions (41%).

### Sample of A&F interventions for application of the evaluation tool

Table 2 (reproduced from the systematic review [26]) describes the sample of critical care A&F intervention studies assessed. The review [26] identified a heterogeneous sample of multicomponent quality improvement interventions involving A&F; eight studies aimed to improve lab test ordering [29–35, 44, 45] and ten studies aimed to improve transfusion ordering [36–45]; two of these studies aimed to improve both practices [44, 45], and one compared two types of A&F interventions [39]. Fifteen of the 17 interventions incorporated one or more additional components, such as education, guidelines, opinion leaders, financial incentives, checklists, or administrative interventions. The plurality of interventions reported providing feedback more than once (53%), in only a written format (41%), with data aggregated at the group level only (41%). Feedback was most often provided to multiple groups of healthcare providers (29%) or physicians only (24%). Heterogeneity of the outcomes precluded meta-analysis; however, overall the majority of interventions reported statistically significant behavior changes in the hypothesized direction. Most studies were judged to be of high risk of bias, due to use of an uncontrolled before/after design, lack of time series analysis, and poor reporting of intervention details, hindering replication.

**Table 2:** Summary of study characteristics^a,d^

Clinical behavior targeted	Number (%) of studies (***n*** = 16)	Country	Number (%) of studies (***n*** = 16)
**Laboratory test ordering**	**8 (50.0%)**	USA	9 (56.3%)
Multiple, miscellaneous or unspecified tests	3 (18.8%)	Canada	2 (12.5%)
ABG	2 (6.3%)	Finland	1 (6.3%)
Lactate and blood cultures	1 (6.3%)	Germany	1 (6.3%)
Superficial cultures	1 (6.3%)	Israel	1 (6.3%)
Blood work	1 (6.3%)	The Netherlands	1 (6.3%)
**Transfusion ordering**	**10 (62.5%)**	Switzerland	1 (6.3%)
RBCs	6 (37.5%)	**Number of sites**	
FP/FFP	3 (18.8%)	Single centre, single ICU study	9 (56.3%)
All (RBC, FFP, platelets, cryoprecipitate)	1 (6.3%)	Single centre, multi-ICU study	4 (25.0%)
**Study design**		Multicentre study	2 (12.5%)
Uncontrolled before after	13 (81.3%)	Single centre, # ICUs unclear	1 (6.3%)
Cluster randomized controlled trial	1 (6.3%)	**Hospital type**	
Controlled clinical trial^b^	1 (6.3%)	Teaching	11 (68.8%)
Controlled before after	1 (6.3%)	Not Reported	3 (18.8%)
**Data collection**		Other: Veteran’s Administration Medical Centre	1 (6.3%)
Prospective	5 (31.3%)	**ICU type**	
Retrospective	4 (25.0%)	Surgical	2 (12.5%)
Mixed	3 (18.8%)	Neonatal	2 (12.5%)
Unclear	4 (25.0%)	Cardiac Surgery	3 (18.8%)
**Funding**		Neurosurgical	1 (6.3%)
Not reported	8 (50.0%)	Medical	1 (6.3%)
Government grant	4 (25.0%)	Mixed Patient Population	2 (12.5%)
Institutional^c^ and non-profit grants	2 (12.5%)	Multiple Types of ICUs	3 (18.8%)
Institutional^c^	1 (6.3%)	Not Specified	2 (12.5%)
No Funding	1 (6.3%)		

*ABG* arterial blood gas, *CP* cryoprecipitate, *FP* frozen plasma, *FFP* fresh frozen plasma, *ICU* intensive care unit, *RBC* red blood cell

^a^Proportions were calculated for the 16 studies, rather than the 17 publications. Totals may be slightly greater or less than 100% due to rounding

^b^The control group was another type of A&F

^c^‘Institution’ refers to both hospitals and academic institutions

^d^Reprinted from *Implementation Science* [26] (Open Access)

### Application of the evaluation tool

**Fig. 1:**
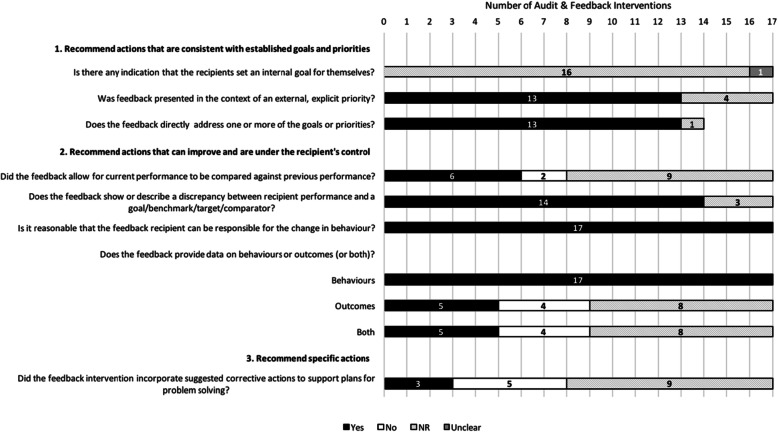
Description of feedback interventions according to the ‘Nature of the Desired Action’ items (*n* = 17 interventions). Note: For items where the total number of interventions is less than 17, the item was rated as ‘Not Applicable’ in the remaining cases

**Fig. 2:**
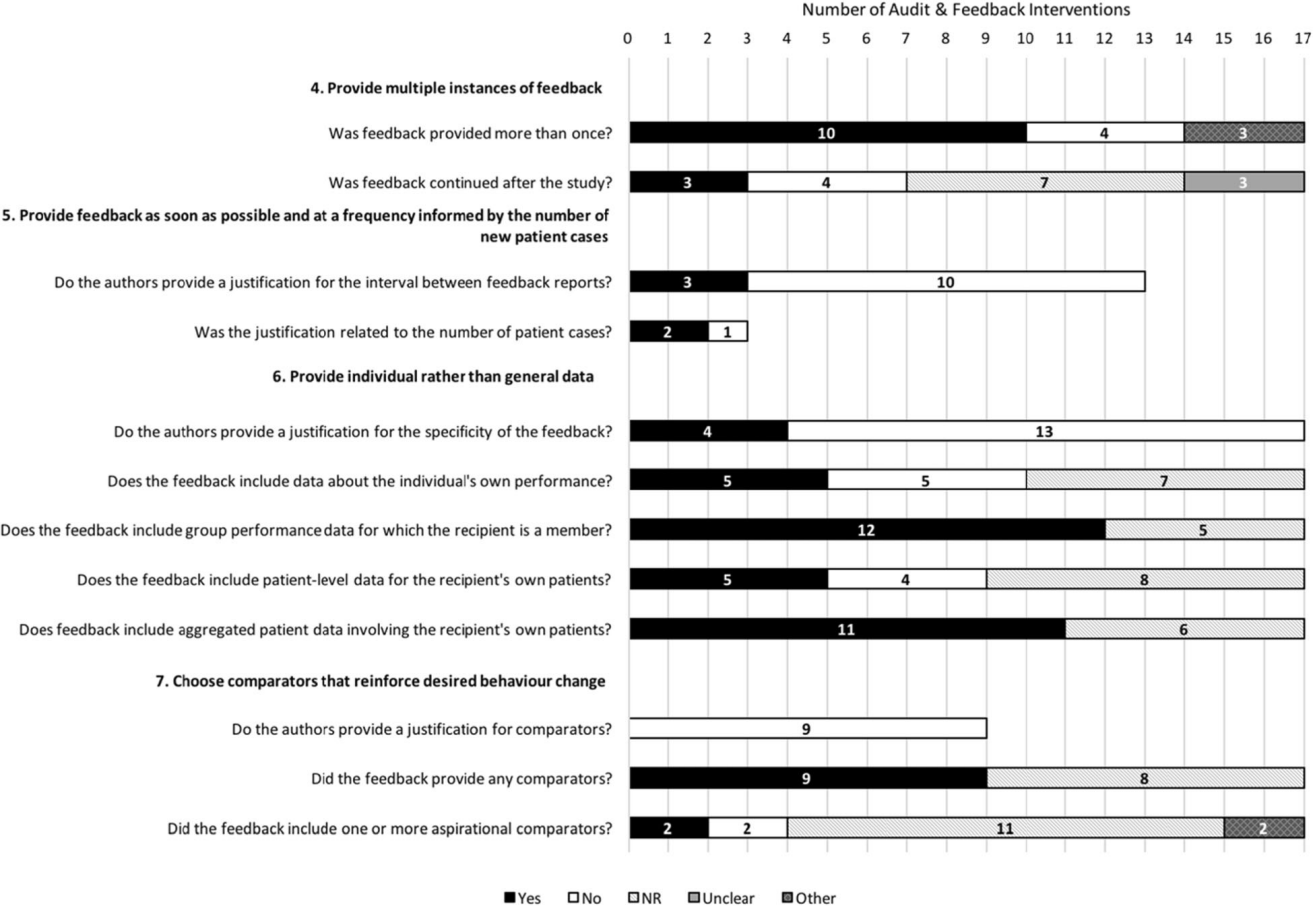
Description of feedback interventions according to the ‘Nature of the Data Available’ items (*n* = 17 interventions). Note: For items where the total number of interventions is less than 17, the item was rated as ‘Not Applicable’ in the remaining cases

**Fig. 3:**
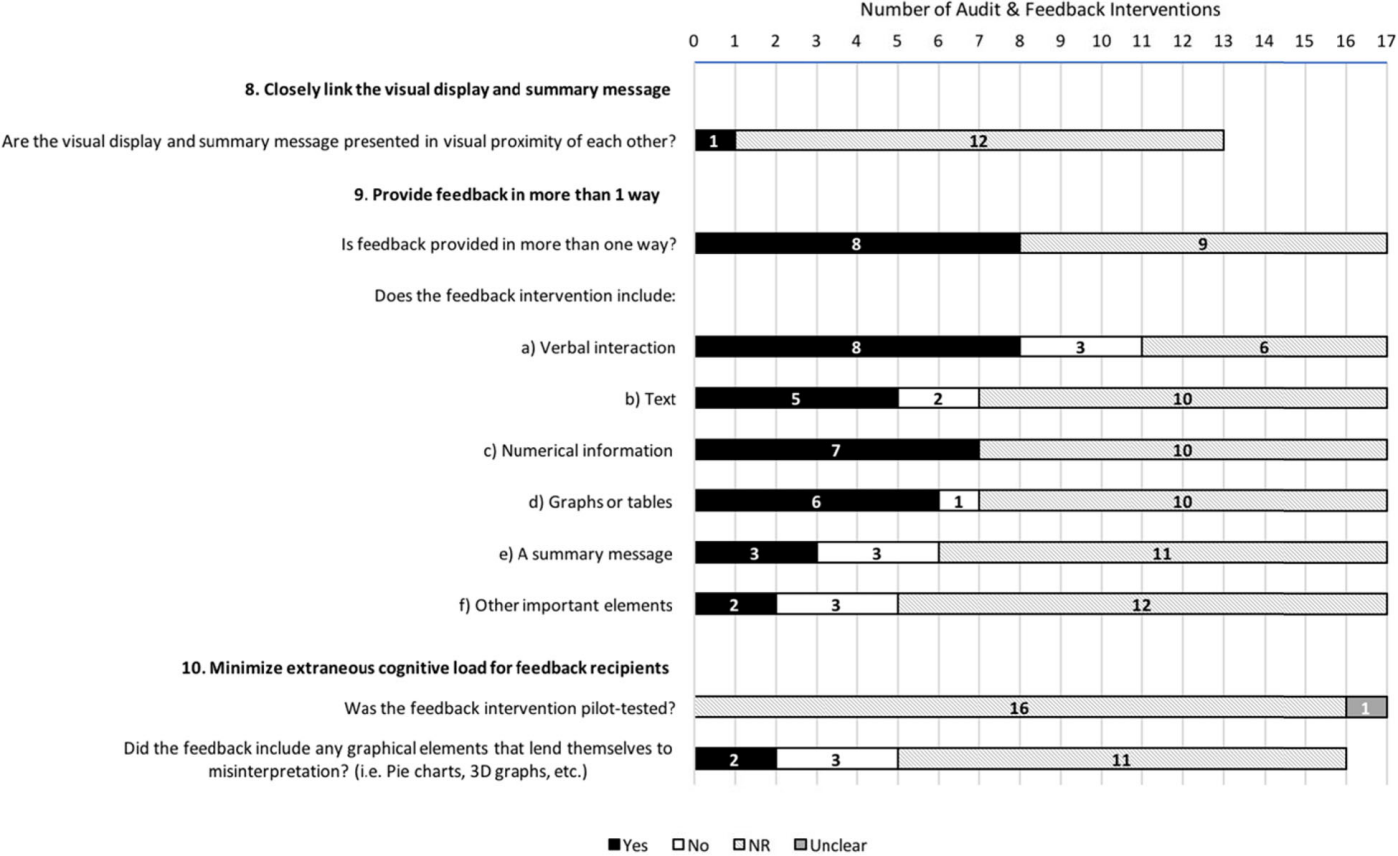
Description of feedback interventions according to the ‘Feedback Display’ items (*n* = 17 feedback interventions). Note: For items where the total number of interventions is less than 17, the item was rated as ‘Not Applicable’ in the remaining cases

**Fig. 4:**
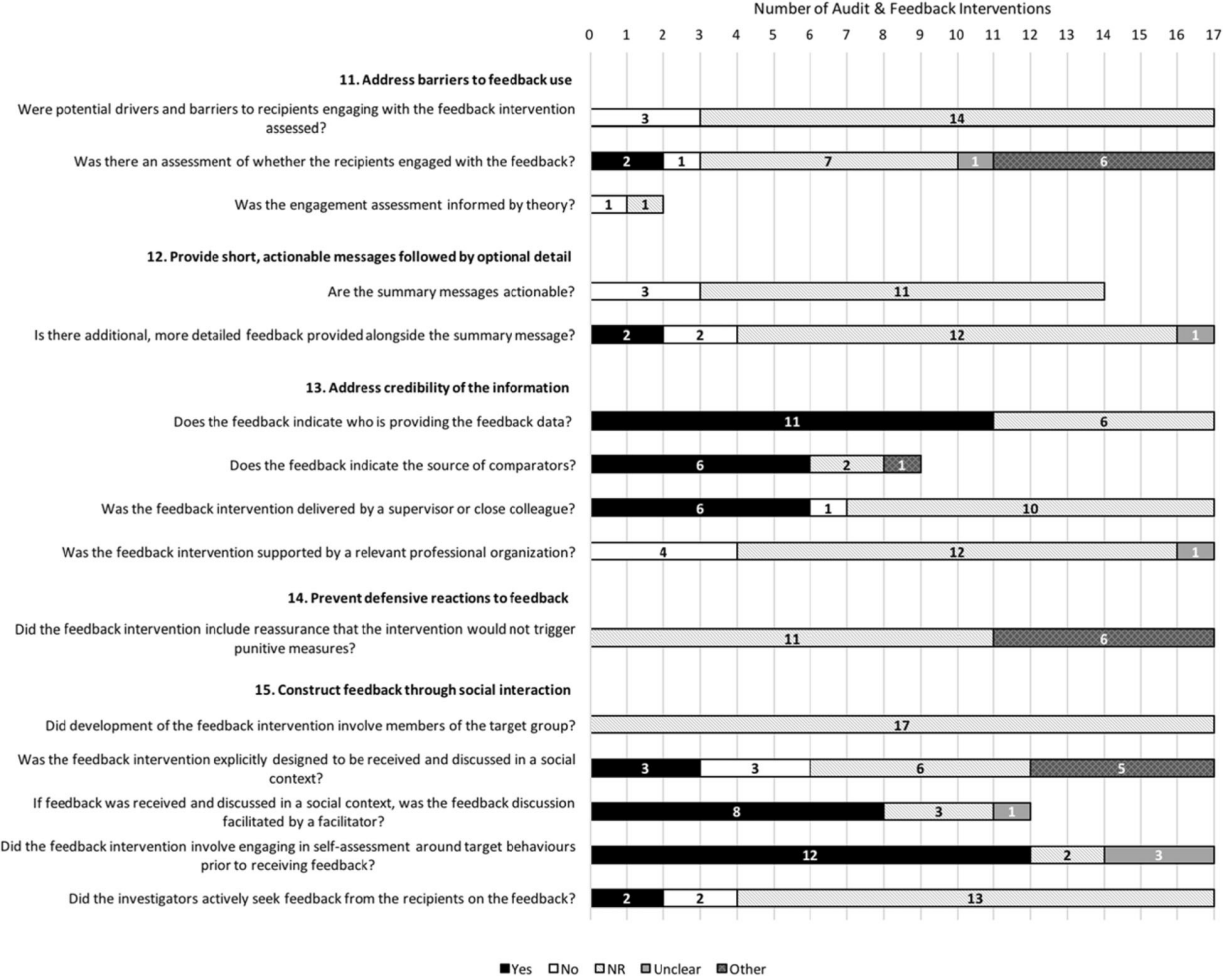
Description of feedback interventions according to the ‘Delivering the Feedback Intervention’ items (*n* = 17 feedback interventions). Note: For items where the total number of interventions is less than 17, the item was rated as ‘Not Applicable’ in the remaining cases

Figures 1, 2, 3, and 4 describe consistency with the 38 ratable items and 3 of the 14 descriptive items (those with a “Yes/No/Not Reported/Unclear/Not Applicable” scale). To enhance clarity, the remaining 11 descriptive items (e.g., *Level at which the priority was set*) have been described in Additional File 5.

### Nature of the desired action

Figure 1 describes adherence to the eight ratable items operationalized from the three “Nature of the Feedback’s Desired Action” suggestions. Descriptions about presenting feedback interventions in the context of external priorities were generally adhered to (13/17), and the feedback generally addressed these priorities (13/17), but information about whether the feedback involved setting of internal goals by recipients was rarely made clear (16/17 rated as not reported; 1/17 unclear). For all interventions (17/17), it was found to be reasonable that the feedback recipient could be responsible for the change in behavior, and most interventions (14/17) showed or described a discrepancy between recipient performance and a goal, benchmark, target or comparator. However, whether the feedback allowed for comparison of current performance against previous performance was adhered to variably (6/17), and few interventions (3/17) explicitly incorporated suggested corrective actions to support plans for problem solving (e.g., action plan, coping strategy, menu of options, etc.).

### Nature of the data available for feedback

Figure 2 describes the ten ratable items and two of the descriptive items derived from the four “Nature of the Data Available for Feedback” suggestions. Though the majority of interventions reported providing feedback more than once (10/17; other (unclear/variable): 3/17), few reported continuing to provide feedback after the study (3/17). Only some of the interventions adhered to including data about the individual’s own performance (5/17) and patient-level data (5/17) (most reported including group level performance data (12/17) and aggregated patient data (11/17)). About half of interventions reported adherence to providing a comparator (9/17); however, few reported including an aspirational comparator (2/17; and other (100% compliance implied but not made explicit): 2/17). Justifications were rarely provided for specificity of the feedback (4/17), choice of comparator(s) (0/9), or the interval between feedback reports (3/13; though 2/3 justifications were related to the number of patient cases).

### Feedback display

Figure 3 describes the sample’s adherence to the four ratable items (and one of the description items) operationalized from the three “Feedback Display” suggestions. Just under half of interventions (8/17) adhered to providing feedback in more than one way; interventions clearly included a verbal feedback component in eight cases, numerical information in seven, graphs or tables in six, text in five, a summary message in three, and other (color coding) in two. However, none of the interventions reported pilot-testing of the feedback (0/17; one intervention unclear). Only one intervention adhered to presenting a visual display and summary message in visual proximity of each other, though in the majority of cases, not enough information was reported to determine this (12/13 not reported; 4/17 not applicable). Of the five ratable cases, two interventions were found to include graphical elements that lend themselves to misinterpretation.

### Delivering the feedback intervention

Figure 4 describes the sample’s adherence to the 16 ratable items operationalized from the five “Delivery of the Feedback” suggestions. Interventions rarely reported conducting barrier/enabler assessments (0/17) or assessments of whether recipients engaged with the feedback (2/17; though 6/17 were rated as “other” [no or not reported, but verbal feedback component]). No interventions reported use of theory to inform such assessments (17/17 not applicable and 0/2, respectively). None of the interventions reported involving members of the target group in the development of the feedback, and none were found to include “actionable” summary messages (0/14, though not reported in 11/14). Few interventions were explicitly designed to be received and discussed in a social context (3/17), though 5/17 were rated as “other” (no explicit statement reported, but feedback was provided in a social context). Furthermore, few actively sought feedback from the recipients (2/17) or provided additional more detailed feedback alongside the summary message (2/17, though not reported in 12/17). However, most interventions did adhere to clearly indicating who was providing the feedback (e.g., provided verbally or through email) (11/17), and most including a comparator clearly indicated the source of the comparator(s) (6/9; and 1/9 “other” [yes, no form but would be obvious; overall institution versus own specific ward]). Although no interventions were found to be supported by a relevant organization or explicitly reported providing reassurance that the intervention would not trigger punitive measures, some were delivered by a supervisor or close colleague (6/17) or reported other methods aiming to reduce defensive reactions (6/17). Most interventions also involved engaging in self-assessment around the target behavior(s) prior to receiving feedback (e.g., an educational session) (12/17), and of those that involved receiving and discussing feedback in a social context, most were facilitated by a facilitator (8/12).

## Discussion

The development of our evaluation tool represents an important step forward in improving A&F interventions. A total of 52 criteria items (38 ratable and 14 descriptive) were operationalized from the 15 suggestions for best practice [4]. To address the uncertainty surrounding the specifics of how best to apply each suggestion, we developed a comprehensive set of items which aimed to capture the various ways in which these suggestions could be employed. This tool allows for assessment of the extent to which A&F interventions adhere to recent guidance for best practice [4]. Future studies may apply this tool to assess how A&F interventions in various settings adhere to these items, as well as whether adoption of these practices improves over time. Moreover, our tool may be used prospectively for the development of A&F interventions, to test the various hypotheses.

Our work to apply this evaluation tool to a sample of critical care related A&F interventions shows that most items are not being consistently implemented or reported across the critical care A&F literature. Of the 38 ratable items, only two were universally applied (*Is it reasonable that the feedback recipient can be responsible for the change in behavior*? and *Does the feedback provide data on behaviors or outcomes* (*or both*)?). This was not particularly surprising as all studies within the sample were published prior to or within the same year as the suggestions for best practice [4], which were hypothesized to be relatively underutilized elements within the existing A&F literature. The results from this study suggest there may be considerable room for improvement in the development and delivery of A&F interventions for laboratory test and transfusion ordering in the critical care setting and point to several theoretical considerations that warrant further study.

We also found that the study details required to assess many items were simply not reported (20/52 items were not reported in the majority of studies). It was especially difficult to assess adherence with the items related to the design and delivery of the feedback, as we were not always able to access an example of the feedback form (had access to 7/17 feedback templates (41%), one form was partial). Better access to feedback form templates may have allowed for more complete extraction of the details necessary for our assessment. Our findings suggest a standardized method for reporting A&F intervention details and readier access to feedback form templates may help to move research in this field forward.

### Use of theory in A&F

There is interest and utility in utilizing theory to improve the design, implementation, and assessment of behavior change interventions, as suggested in the Medical Research Council’s guidance [46, 47]. A priori predictions of mechanisms of action for complex interventions through consideration of relevant theories can facilitate a better understanding of why an intervention is or is not successful [48–50]. Recent analyses of the A&F literature (the 140 studies included in the Cochrane systematic review) [48], as well as the more general implementation literature (guideline implementation) [51], however, have revealed low rates of reported theory use. A review assessing the use of theory across Cochrane systematic review A&F interventions found theory was mentioned in only 14% of studies, and only 9% of them referenced theory in terms of A&F design [48]. Researchers may have difficulty selecting theories for application to their interventions and studies, due to the lack of consensus and, until recently, lack of guidance on how best to choose from numerous theories [52–54]. To synthesize theoretically informed guidance for A&F developers, Brehaut and colleagues conducted interviews with theory experts and drew from team experience and systematic reviews [4]. Our finding that many of these theoretically informed suggestions are underutilized in the existing critical care A&F literature is therefore in line with these previous studies. Below we’ve described several key suggestions for which our sample showed low consistency, to highlight priorities for future research.

### Underutilized suggestions in critical care A&F

Counter to Brehaut et al.’s suggestion to “provide multiple instances of feedback” [4], some interventions (4/17) only provided feedback once, while others did not clearly report whether feedback was provided more than once or provided feedback variably (e.g., only if an order was placed inappropriately) (3/17). This finding is important because providing feedback more than once allows for a cyclical process whereby the recipient first receives feedback on their behavior (and potentially suggestions on how to improve) [4]. If allowed the chance to change their behavior, upon receiving feedback again, individuals may gauge whether their efforts were successful or not. Without iterative feedback, recipients may not be able to determine their progress on whether their efforts were successful. As it is suggested that individuals are unable to accurately assess their own performance, this is an important part of the feedback loop [1, 4, 55].

Several A&F interventions (7/17, 41%) only clearly reported presenting data aggregated at the group level. Brehaut et al.’s recent guidance suggests feedback provide data at the individual level, to dissuade discounting of the data [4]. However, as noted in one study, it may be difficult to “assign” orders to individual healthcare providers if the decision is made by the team [38]. A previous meta-analysis also found a combination of group and individual data to result in a larger effect size than either type alone [56]. It may therefore be of interest to further assess whether providing both individual and group level data is more effective in team-based settings such as the ICU.

While none of the studies reported providing reassurance that the feedback intervention would not result in punitive measures, studies for six of the interventions did report the incorporation of other aspects (e.g., providing both positive and negative feedback, providing group data to avoid singling out individuals, using non-punitive wording, etc.) that were consistent with the suggestion to aim to “Prevent defensive reactions to feedback” [4]. This is an important component, as previous qualitative work has identified that providers may have initial defensive reactions to negative feedback [57, 58]. It is also imperative to note that our criteria item represents only one way of potentially preventing defensive reactions, the effectiveness of which should still be tested. Further work is required to elucidate methods on how best to avoid negative reactions to feedback.

Other areas of poor adherence included the lack of reported use of elements such as piloting of the feedback form, involvement of key stakeholders, barrier and engagement assessments, goal setting, and action and coping plans. Due to a lack of reporting, it is unclear whether low adherence may simply represent a reporting issue, or if these steps are not being taken. If these elements are not being incorporated, it would be valuable to assess whether incorporation of these elements in A&F studies helps to improve behavior change in the critical care setting. Involving stakeholders in the development process may also help to identify priorities and appropriate modalities through which to provide feedback. Previous qualitative work has found that ICU specialists feel A&F to be a “fragmented or discontinuous communication,” “often not actionable,” and have noted that the audit process can “[lack] transparency and credibility” [57]. Engaging stakeholders throughout the development of the feedback may also help to ensure providers feel a part of the process and that the feedback provided is useful and positively received. Further, as laboratory test and transfusion ordering are likely habitual behaviors, use of supports such as action or coping plans may be especially pertinent, because it’s hypothesized that these plans can help to form new habits [12, 59].

### Strengths and limitations

A limitation of our study is the potential for lack of reporting on key details in the intervention descriptions available to us. As demonstrated by Colquhoun et al., the reporting quality of A&F intervention details varies [60]. Since the majority of studies in our sample used multiple intervention components, space limits may have especially inhibited reporting of such details. As varied reporting was anticipated, we aimed to counter this limitation by contacting the study authors to obtain further details and request an example of the feedback form used during the study; having access to the feedback form allows for more complete coding. Another limitation of our tool is that without access to feedback examples, a number of the developed criteria items are difficult or not possible to rate (e.g., items relating to the feedback design). An important way to move this literature forward and ensure that A&F interventions improve is thus to make these documents available. We also note that there are limitations to the development approach taken. Though this work builds on guidance developed through a comprehensive approach (systematic reviews, interviews, team experience), it was still limited to this set of recommendations. Thus, there may still be additional important elements for A&F not captured. Furthermore, as our sample was limited to the critical care setting, it is also unclear whether our results would have been similar across different patient populations or stakeholder groups. Application of our evaluation tool to different settings, and by external users, will be a valuable area for future study, and important in ensuring generalizability of the tool. Further psychometric testing will also be required in future work to assess construct, content and criterion validity [61]. Due to the variation in reporting of intervention details, limited access to feedback templates, and above mentioned limitations, the current evaluation tool, which we have tentatively named REFLECT-52 (REassessing audit & Feedback interventions: a tooL for Evaluating Compliance with suggested besT practices), cannot claim to quantitatively assess quality of feedback display. However, currently, we see this tool as a way to help developers *reflect* on their A&F interventions and consider whether they may be able to improve their A&F. We hope to continue to streamline and iteratively improve this tool over time, by working to incorporate new findings and guidance for A&F development.

## Conclusions

We developed a theory-informed 52-item tool for assessing the degree of concordance of A&F interventions with best practice recommendations and applied it to A&F in critical care. Within critical care, only two items were adhered to by all studies. Our evaluation tool provides a potential way forward to help improve reporting of A&F interventions, for assessing their concordance with agreed best practice and to inform the development of improved A&F interventions.

## Supplementary Information

**Additional File 1.** Consolidated criteria for reporting qualitative studies (COREQ) checklist.

**Additional File 2.** Cohen’s Kappa Calculation.

**Additional File 3.** Full evaluation tool, including response scales and anchors.

**Additional File 4.** PRISMA flow diagram of the study selection.

**Additional File 5.** Assessment of remaining descriptive items.

## Data Availability

The datasets used and/or analyzed during the current study are available from the corresponding author on reasonable request; however, the data and materials pertaining to individual participants will not be shared to protect privacy. Coding tools are also available from authors upon request.

## Abbreviations

A&F: Audit and feedback
ICU: Intensive care unit
