# Supplementary material for: How well do critical care audit and feedback interventions adhere to best practice? Development and application of the REFLECT-52 evaluation tool
Source: Implement Sci. 2021 Aug 17;16:81. doi: 10.1186/s13012-021-01145-9 (PMC8369748; doi:10.1186/s13012-021-01145-9)
Supplement: Supplementary file 1 — Additional File 1. Consolidated criteria for reporting qualitative studies (COREQ) checklist. [file 13012_2021_1145_MOESM1_ESM.docx]

**Additional File 1: Consolidated criteria for reporting qualitative studies (COREQ) checklist**

| **No. Item** | **Guide Questions/Descriptions** | **Reported on Page #** |  |
| --- | --- | --- | --- |
| **Domain 1. Research team and reflexivity**  **Personal Characteristics** | | | |
| Interviewer/facilitator | Which author/s conducted the interview or focus group? | N/A  Pilot: MF, MP  Data extraction: MF, EP  7-8 |  |
| Credentials | What were the researcher’s credentials? E.g. PhD, MD | MF/EP: MSc. Candidate  MP: BA Candidate |  |
| Occupation | What was their occupation at the time of the study? | MF/EP: MSc. Epidemiology student  MP: Research assistant |  |
| Gender | Was the researcher male or female? | MF/MP: Female  EP: Male |  |
| Experience and training | What experience or training did the researcher have? | MF/MP: Developed and piloted evaluation tool  EP: Trained by MF |  |
| **Relationship with participants** | | |  |
| Relationship established | Was a relationship established prior to study commencement? | N/A |  |
| Participant knowledge of the interviewer | What did the participants know about the researcher? e.g. personal goals, reasons for doing the research | N/A |  |
| Interviewer characteristics | What characteristics were reported about the interviewer/facilitator? e.g. Bias, assumptions, reasons and interests in the research topic | N/R |  |
| **Domain 2: Study Design**  **Theoretical framework** | | | |
| Methodological orientation and Theory | What methodological orientation was stated to underpin the study? e.g. grounded theory, discourse analysis, ethnography, phenomenology, content analysis | 4-7 |  |
| **Participant selection** | | | |
| Sampling | How were participants selected? e.g. purposive, convenience, consecutive, snowball | 6 |  |
| Method of approach | How were participants approached? e.g. face-to-face, telephone, mail, email | 6 |  |
| Sample size | How many participants were in the study? | 9 |  |
| Non-participation | How many people refused to participate or dropped out?  Reasons? | 9 |  |
| **Setting** | | | |
| Setting of data collection | Where was the data collected? e.g. home, clinic, workplace | N/A |  |
| Presence of non-participants | Was anyone else present besides the participants and researchers? | N/A |  |
| Description of sample | What are the important characteristics of the sample? e.g. demographic data, date | 9-10; Table 2 |  |
| **Data collection** | | | |
| Interview guide | Were questions, prompts, guides provided by the authors?  Was it pilot tested? | Additional File 1  7 |  |
| Repeat interviews | Were repeat interviews carried out? If yes, how many? | N/A |  |
| Audio/visual recording | Did the research use audio or visual recording to collect the data? | N/A |  |
| Field notes | Were field notes made during and/or after the interview or focus group? | N/A |  |
| Duration | What was the duration of the interviews or focus group? | N/A |  |
| Data saturation | Was data saturation discussed? | N/A |  |
| Transcripts returned | Were transcripts returned to participants for comment and/or correction? | N/A |  |
| **Domain 3: Analysis and findings**  **Data analysis** | | | |
| Number of data coders | How many data coders coded the data? | 7-8 |  |
| Description of the coding tree | Did authors provide a description of the coding tree? | Additional File 1 |  |
| Derivation of themes | Were themes identified in advance or derived from the data? | N/A |  |
| Software | What software, if applicable, was used to manage the data? | 8 |  |
| Participant checking | Did participants provide feedback on the findings? | N/A |  |
| **Reporting** | | | |
| Quotations presented | Were participant quotations presented to illustrate the themes / findings? Was each quotation identified? e.g. participant number | N/A |  |
| Data and findings consistent | Was there consistency between the data presented and the findings? | Figures 1-4  10-16 |  |
| Clarity of major themes | Were major themes clearly presented in the findings? | 13-16 |  |
| Clarity of minor themes | Is there a description of diverse cases or discussion of minor themes? | 10-13, 15-16 |  |
