# Supplementary material for: How well do critical care audit and feedback interventions adhere to best practice? Development and application of the REFLECT-52 evaluation tool
Source: Implement Sci. 2021 Aug 17;16:81. doi: 10.1186/s13012-021-01145-9 (PMC8369748; doi:10.1186/s13012-021-01145-9)
Supplement: Supplementary file 2 — Additional File 2. Cohen’s Kappa Calculation. [file 13012_2021_1145_MOESM2_ESM.pdf]

Additional File 2: Cohen's Kappa Calculation

Overall Agreement		Madison				
		Yes	No	Unclear	N/A	Total
Maria	Yes	108	9	16	4	137
	No	21	183	19	22	245
	Unclear	9	20	22	2	53
	N/A	0	2	3	24	29
	Total	138	214	60	52	464

Total Agreement (n) = 108+ 183+22+24= 337

Total Agreement (%) = (337/464) x 100 = 72.6%

Code	Calculation	Expected Frequency
Yes	(137 x 138)/464	40.7
No	(214 x 245)/464	113.0
Unclear	(60 x 53)/464	6.9
N/A	(52 x 29)/464	3.25
Sum	40.7+ 113.0 + 6.9 +3.25	163.85

Cohen's Kappa

K = (Σ agreement -Σ expected frequency)/(N-Σ expected frequency)

K= (337-163.85)/(464-163.85)

K= 0.58
