# Supplementary material for: How well do critical care audit and feedback interventions adhere to best practice? Development and application of the REFLECT-52 evaluation tool
Source: Implement Sci. 2021 Aug 17;16:81. doi: 10.1186/s13012-021-01145-9 (PMC8369748; doi:10.1186/s13012-021-01145-9)
Supplement: Supplementary file 3 — Additional File 3. Full evaluation tool, including response scales and anchors. [file 13012_2021_1145_MOESM3_ESM.docx]

**Additional File 3: Evaluation Tool Criteria Items, Response Scales and Anchors Developed from Brehaut et al.’s 15 Suggestions**^1^

| **Description** | **Criteria Item** | **Response Scale**^[[1]](#footnote-1)^ | **Anchor** |
| --- | --- | --- | --- |
| **Suggestion #1: Recommend actions that are consistent with established goals and priorities** | | | |
| 1.1 Goal | Is there any indication that the recipients set an internal goal for themselves (i.e. a specific, numerical target/threshold)? | Yes, No, Not Reported, Unclear | Code as **'Yes'** if the feedback component of the intervention involved the recipients setting an internal goal for themselves. **Example:** The feedback component of the intervention included peer discussions where individual goals were set and discussed.  Code as **'No'** if the feedback component of the intervention did not involve the recipients setting an internal goal (and the feedback form or additional details are available to confirm this).  Code as **'Not reported'** if there are no details in the text regarding whether the feedback component of the intervention involved internal goal setting by the recipients (and the feedback form/additional details are not available).  Code as **‘Unclear’** if the text contains an ambiguous statement and the feedback form/additional details are not available to confirm. |
| 1.2 Priority | Was feedback presented in the context of an external, explicit priority? | Yes, No, Not Reported, Unclear | Code as **'Yes'** if the feedback is given in the context of a guideline or initiative. **Note:** This guideline or initiative should be made clear to the feedback recipients (i.e. is discussed on the feedback form, OR the article describes that the recipients were aware of the guideline/initiative).  Code as **'No'** if there is no indication that feedback was given in the context of a guideline or initiative. **Example:** The authors generally discuss guidelines or the need to change a behaviour in the introduction, but there is no indication of this priority on the feedback form.  Code as **'Not reported'** if there are no details in the text regarding whether the feedback intervention involved an external, explicit priority (and the feedback form/additional details are not available).  Code as **‘Unclear’** if the text contains an ambiguous statement and the feedback form/additional details are not available to confirm.  **Note:** There could potentially be more than one priority (i.e. A provincial guideline and a hospital initiative). |
| 1.3 Priority Level | If yes, at what level was the priority set? | National/Federal, Provincial/State, Municipal, Institutional, Departmental, Individual-healthcare providers, Individual- researchers, Other (Please specify), N/A (No discussion of an explicit priority), Unclear, Not reported |  |
| 1.4 Link to feedback | Does the feedback directly address one or more of the goals or priorities? | Yes, No, Not Reported, Unclear, N/A (no goal/priority) | Code as **'Yes'** if the feedback corresponds with either: one of the goals set by recipients or one of the external priorities presented. **Example:** Feedback is presented in the context of a hospital initiative to reduce inappropriate transfusions and the feedback provides information on the number of transfusions which were inappropriate, along with suggestions on how to reduce inappropriate orders.  Code as **'No'** if the feedback does not correspond with one of the goals set by recipients or one of the external priorities presented. **Example:** Feedback is presented in the context of a provincial guideline to reduce inappropriate laboratory tests, but the feedback to recipients only provides information on their laboratory expenditure (i.e. there is no information on the appropriateness of the lab tests).  Code as **'Not reported'** if there are no details in the text and the feedback form/additional details are not available.  Code as **‘Unclear’** if the text contains an ambiguous statement and the feedback form/additional details are not available to confirm.  Code as **'N/A (no goal/priority)'** if the feedback recipients do not set internal goals for themselves and the feedback is not presented in the context of a priority. |
| **Suggestion #2: Recommend actions that can improve and are under the recipient’s control** | | | |
| 2.1 Previous performance | Was feedback on performance provided to allow current performance to be compared against previous performance? | Yes, No, Not Reported, Unclear | Code as **'Yes'** if the feedback shows an individual's performance over time (i.e. performance data from at least two time points).  Code as **'No'** if the feedback only shows performance from one time point.  Code as **'Not reported'** if there are no details in the text and the feedback form/additional details are not available.  Code as **‘Unclear’** if the text contains an ambiguous statement and the feedback form/additional details are not available to confirm. |
| 2.2 Discrepancy | Does the feedback (or the description of the feedback) describe or show a discrepancy between recipient performance and the goal/ benchmark/ target/comparator? | Yes, No, Not Reported, Unclear | Code as **'Yes'** if the feedback included at least one comparator (including 'own previous performance') and the discrepancy between these two performance levels was made clear (i.e. the difference is noted, a bar graph is displayed etc.). Can also code as **‘Yes’** if the feedback clearly relays that recipients need to change their behaviour (i.e. they were identified in a review for inappropriate transfusions). Can also code as ‘**Yes’** if the feedback displays compliance or non-compliance on a scale of 0-100% (this implies that there is a discrepancy in behaviour).  Code as **'No'** if no comparators were provided, or if a comparator was provided and the discrepancy was not made clear (i.e. the performance of the individual is displayed on one page, and the performance of the comparator is displayed on another; or the feedback shows a recipient’s performance over time and it is not obvious whether they are improving or not (no target or benchmark)).  Code as **'Not reported'** if there are no details in the text and the feedback form/additional details are not available (i.e. the article does not include an example of the feedback form and from the description of the intervention it is not clear whether the performance levels were displayed on the same graph or not).  Code as **‘Unclear’** if the text contains an ambiguous statement and the feedback form/additional details are not available to confirm. |
| 2.3 Control | Is it reasonable that the feedback recipient can be responsible for the change in behaviour? | Yes, No, Not Reported, Unclear | **Note:** The recipient does not need to be directly responsible. Code as **'Yes'** if the recipient can make a difference in the behaviour being fed back, either directly (i.e. it is their behaviour that needs to change) or indirectly (i.e. they are to discuss with/train the individuals whose behaviour needs to change).  Code as **'No'** if the feedback recipient cannot be responsible for the change in behaviour. **Example:** feedback on physician test ordering is provided to the CEO of the hospital.  Code as **'Not reported'** if there are no details in the text and the feedback form/additional details are not available.  Code as **‘Unclear’** if the text contains an ambiguous statement and the feedback form/additional details are not available to confirm whether the recipient can make a difference in the behaviour being fed back. |
| 2.4 Outcome Type | Does the feedback provide data on behaviours, outcomes, or both? | For each, answer: Yes, No, Not Reported, Unclear | Behavioural data includes process measures such as prescribing habits, test ordering, etc. Outcome data includes patient outcomes such as patient blood pressure, mortality, length of stay etc.  Code as **‘Yes’** if clearly present.  Code as **‘No’** if clearly absent.  Code as **'Not reported'** if there are no details in the text and the feedback form/additional details are not available.  Code as **‘Unclear’** if the text contains an ambiguous statement and the feedback form/additional details are not available to confirm. |
| **Suggestion #3: Recommend specific actions** | | | |
| 3.1 Corrective Actions | Did the feedback intervention incorporate suggested corrective actions to support plans for problem solving? For example: action plans, coping strategies, a menu of options, etc. | Yes, No, Not Reported, Unclear | **Note:** Actions may be mandated or developed in a group setting. Code as **'Yes'** if the feedback intervention provided an action plan, a coping strategy, a menu of options, or facilitated recipients in developing a plan. **Example:** The feedback indicated that a physician's actions were non-compliant with the guidelines and provided a menu of options to proceed [i.e. “(1) rescheduling the patient sooner, (2) marking the clinic encounter form... to suggest the physician perform the indicated preventative care on the patient’s next scheduled visit, (3) indicating the protocol was not applicable for this patient (but the physician agrees, in principle, with the protocol), (4) stopping this reminder (the physician disagrees with the protocol), and (5) pulling the patient’s chart for review”].^2^  Code as **'No'** if the feedback intervention did not provide corrective actions to support plans for problem solving.  Code as **'Not reported'** if there are no details in the text and the feedback form/additional details are not available.  Code as **‘Unclear’** if the text contains an ambiguous statement and the feedback form/additional details are not available to confirm whether the feedback intervention included corrective actions to support plans for problem solving. |
| **Suggestion #4: Provide multiple instances of feedback** | | | |
| 4.1 Number of feedback reports | Was feedback (for a given behaviour) provided more than once? | Yes, No, Not Reported, Unclear, Other | **Note:** One feedback report with multiple time-points constitutes receiving feedback once. Three feedback reports, each on a different clinical behaviour^3^ constitutes receiving feedback once for a given behaviour.  Code as **'Not reported'** if there are no details in the text and the feedback form/additional details are not available.  Code as **‘Unclear’** if the text contains an ambiguous statement and the feedback form/additional details are not available to confirm. |
| 4.2 Sustained? | Did recipients continue to receive feedback on their performance after the study was completed? | Yes, No, Not Reported, Unclear | **Note: This is a reporting issue.** Code as **'Yes'** if the authors explicitly state that feedback continued after completion of the study.  Code as **'No'** if there is no indication that feedback was provided beyond the study period.  Code as **'Not reported'** if there are no details in the text.  Code as **‘Unclear’** if the text contains an ambiguous statement and it is unclear whether the feedback initiative was continued beyond the study. **Example:** The authors state that a larger scale program was being prepared, but it is unclear whether or not this program was actually put in place.^3^ |
| **Suggestion #5: Provide feedback as soon as possible and at a frequency informed by the number of new patient cases** | | | |
| 5.1 Age of the data | What is the average age of the data (i.e. interval between the clinical encounter and delivery of the feedback)? | Describe (i.e. Days, Weeks, Months, Years, Not Reported, Unclear) | **Example 1:** If feedback reports are provided weekly, the average age of the data is 'days'. If feedback reports are provided monthly, the average age of the data is 'weeks'. If feedback reports are provided annually, the average age of the data is 'months', etc.  **Example 2:** If feedback data are collected for one month, and the feedback is provided 3 months after the data collection period, the average age of the data is ‘months’.  Code as **'Not reported'** if there are no details in the text and the feedback form/additional details are not available.  Code as **‘Unclear’** if the text contains an ambiguous statement and the feedback form/additional details are not available to confirm. |
| 5.2 Interval | If feedback was provided more than once, what was the time interval between the receipt of feedback reports? | Describe (i.e. Hours, Days, Weeks, Months, Years, Variable, Not Reported, Unclear, N/A) | **Example:** An interval of 1 week ='days', an interval of 1 month = 'weeks', an interval of 1 year= 'months' etc. |
| 5.3 Justification- Interval | Do the authors of the study provide a justification for the interval between feedback reports? | Yes, No, Unclear, N/A | **Note: This is a reporting issue.** Code as **'Yes'** if the authors state a reason for choosing the interval between feedback reports. This may include a statement in the introduction stating that a certain interval has been effective in the past.  Code as **'No'** if the authors do not provide a reason for choosing the interval between feedback reports.  Code as **'Unclear'** if it is not clear whether the authors have provided a sufficient justification.  Code as **‘N/A’** if feedback was only provided once. |
| 5.4 Justification related to the number of patients? | Was the justification for the interval between feedback reports related to the number of patient cases? | Yes, No, Unclear, N/A | **Note: This is a reporting issue.** Code as **'Yes'** if the justification is related to the number of patient cases. **Example:** To avoid alert fatigue, the authors explain that feedback was only provided quarterly, as physicians only see a few of these patients every month.^1^  Code as **'No'** if the justification is not related to the number of patient cases.  Code as **'Unclear'** if it is unclear whether the justification is related to the number of patient cases.  Code as **'N/A'** if no justification was given. |
| **Suggestion #6: Provide individual rather than general data** | | | |
| 6.1 Individual performance | Was feedback given about the individual's own performance? | Yes, No, Not Reported, Unclear | **Note:** This question does not apply to aggregate level data for a group of which the recipient is a member of; see next question. Code as **'Yes'** if feedback is about an individual's own performance. **Example:** Dr. X receives feedback on his own performance (i.e. The number of B12 tests he ordered).  Code as **'No'** if only group performance data is given.  Code as **'Not reported'** if there are no details in the text and the feedback form/additional details are not available.  Code as **‘Unclear’** if the text contains an ambiguous statement and the feedback form/additional details are not available to confirm whether individual performance data was given. |
| 6.2 Group performance | Was feedback about the performance of a group of which the recipient is a member? | Yes, No, Not Reported, Unclear | Code as **'Yes'** if feedback provides performance data for a group of which the recipient is a member of. **Example:** Dr. X receives feedback on the number of B12 tests ordered by his entire practice.  Code as **'No'** if no group performance data is given.  Code as **'Not reported'** if there are no details in the text and the feedback form/additional details are not available.  Code as **‘Unclear’** if the text contains an ambiguous statement and the feedback form/additional details are not available to confirm whether group level performance data was provided. |
| 6.3 Group level | What is the level of the group? | Describe: Unit, Department, Practice, Hospital, Region, Province, Other (please specify), N/A, Not Reported | Code as **‘N/A’** if previous question (6 b) Group performance) was coded as ‘N/R’. |
| 6.4  Individual data | Did feedback include patient-level data for the recipient's own patients? | Yes, No, Not Reported, Unclear | Code as **'Yes'** if feedback provided individual patient data. **Example:** Dr. Y receives feedback on the number of blood tests she ordered for each individual patient over the past week.  Code as **'No'** if feedback does not provide patient-level data. **Note:** Code as ‘No’ if individual patient data is plotted on a graph, but no identifying information is provided to the recipient (e.g. they are not able to distinguish which data belongs to which patient).  Code as **'Not reported'** if there are no details in the text and the feedback form/additional details are not available.  Code as **‘Unclear’** if the text contains an ambiguous statement and the feedback form/additional details are not available to confirm whether patient level data was provided. |
| 6.5  Aggregated data | Did feedback include aggregated patient data involving recipient's own patients? | Yes, No, Not Reported, Unclear | **Note:** This question applies to any situation (e.g. individual performance level feedback, group performance level feedback, or both).    Code as **'Yes'** if feedback provides aggregated patient data. **Example:** Dr. Y receives feedback on the average number of blood tests she ordered per patient over the past month. OR, Dr.Y receives feedback on the average number of blood tests ordered by her practice over the past month.  Code as **'No'** if feedback does not provide aggregated data (e.g. the feedback only shows data for each individual patient).  Code as **'Not reported'** if there are no details in the text and the feedback form/additional details are not available.  Code as **‘Unclear’** if the text contains an ambiguous statement and the feedback form/additional details are not available to confirm whether aggregated data was provided. |
| 6.6  Justification- Specificity | Do the authors give a justification for the specificity of the feedback (or a reason for the level of data presented)? | Yes, No, Unclear | **Note: This is a reporting issue.** Code as **'Yes'** if the authors provide a justification for either the specificity of the feedback or feedback data. **Example:** We chose to provide individual performance data, as this has been shown to be more effective than group performance data, as per a recent study... etc.  Code as **'No'** if no justification is given for either the specificity of the feedback or the feedback data.  Code as **'Unclear'** if it is unclear whether the authors have provided a reason or not. |
| **Suggestion #7: Choose comparators that reinforce desired behaviour change** | | | |
| 7.1 Comparator? | Did the feedback provide any comparators? | Yes, No, Not Reported, Unclear | **Note:** **Thresholds to define appropriateness (e.g. a transfusion trigger of 8 g/dL) do not qualify as comparators.**  Code as **'Yes'** if recipients were given at least one clear comparator.  Code as '**No'** if recipients were not given a clear comparator.  Code as **'Not reported'** if there are no details in the text and the feedback form/additional details are not available.  Code as **‘Unclear’** if the text contains an ambiguous statement and the feedback form/additional details are not available to confirm. |
| 7.2 Number of Comparators | How many comparators were provided? | 1, 2, 3, More than 3, None, Not Reported, Unclear, Other (describe as needed) | **Note:** A comparator may be a target, benchmark, peer performance or self-comparator if there is a direct comparison to self at different time points. |
| 7.3 Describe | Describe all that apply. | Own previous performance, Other's performance, Benchmark/ Standardized Guideline/Target, Other, Not Reported, Unclear | If 'Other', describe. |
| 7.4  Aspirational Comparator | Does feedback include one or more aspirational comparators (as opposed to average performance comparators)? | Yes, No, Not Reported, Unclear, Other | **Note: Mean/average level comparators do not qualify. Thresholds to define appropriateness (e.g. a transfusion trigger of 8 g/dL) do not qualify as comparators.**  Code as **'Yes'** if the feedback provided a comparator or target based on a high percentile (i.e. the performance of the top 10%) or a standard.  Code as **'No'** if the feedback did not provide a comparator, or if the feedback provided a mean/average level target.  Code as **'Not reported'** if there are no details in the text and the feedback form/additional details are not available.  Code as **‘Unclear’** if the text contains an ambiguous statement and the feedback form/additional details are not available to confirm if the feedback provided a comparator/target and it is unclear whether it is based on a high percentile. |
| 7.5  Justification- Comparators | Do the authors provide a justification for which comparators were used? | Yes, No, Unclear, N/A | **Note: This is a reporting issue.** Code as **'Yes'** if the authors provide a reason for choosing a certain comparator. This may include a statement in the introduction noting the effectiveness of a comparator. **Example:** "Performance feedback, especially with *comparison to peers*, is another effective method of improving compliance with clinical practice guidelines. Although not enough is known about the optimal characteristics of feedback, *comparison with an 'achievable benchmark of care'* appears promising."^4^  Code as **'No'** if no clear reason is given for choosing a comparator.  Code as **'Unclear'** if it is unclear whether the authors provided a reason or not.  Code as **'N/A'** if no comparator is provided. |
| **Suggestion #8: Closely link the visual display and summary message** | | | |
| 8.1 Linking of feedback elements | Are the visual display and the summary message presented in visual proximity of each other? | Yes, No, Not Reported, Unclear, N/A | Code as **'Yes'** if there are no obvious issues with how the feedback elements are linked.  Code as **'No'** if there are any obvious issues with how the feedback elements are linked.  **Example:** 1) The summary message for a graph is shown on a different page than the graph.^1^ **2)** The summary message and visual display give conflicting information i.e. the summary message indicates the physician needs to increase their number of referrals, but the visual display shows that they have more referrals than the top 10% benchmark.^1^  Code as **'Not reported'** if there are no details in the text and the feedback form/additional details are not available.  Code as **‘Unclear’** if the text contains an ambiguous statement and the feedback form/additional details are not available to confirm.  Code as **‘N/A’** if either the summary message or visual display is missing. |
| **Suggestion #9: Provide feedback in more than one way** | | | |
| 9.1 >1 way? | Was feedback provided in more than one way? | Yes, No, Not Reported, Unclear | Based on factors assessed in the next item (9b) was feedback provided in more than one way? |
| 9.2 Format | Does the feedback intervention include: 1) Verbal interaction; 2) Text; 3) Numerical information; 4) Graphs or tables; 5) A summary message; 6) Other important elements (please specify)? | Provide an answer for each item: Yes, No, Not Reported, Unclear | Code as **‘Yes’** if clearly present.  Code as **‘No’** if clearly absent.  Code as **'Not reported'** if there are no details in the text and the feedback form/additional details are not available.  Code as **‘Unclear’** if the text contains an ambiguous statement and the feedback form/additional details are not available to confirm. |
| **Suggestion #10: Minimize extraneous cognitive load for feedback recipients** | | | |
| 10.1 Pilot | Was the feedback intervention pilot-tested? | Yes (With target population), Yes (With non-target population), No, Not Reported, Unclear | **Note: This is a reporting issue.** Code as **'Yes'** if the article indicates that the feedback component of the intervention was pilot-tested; indicate whether or not the pilot took place with the target population.  Code as **'No'** if there is no indication that the feedback component of the intervention was pilot-tested.  Code as **'Not reported'** if there are no details in the text and additional details from the author are not available.  Code as **‘Unclear’** if the text contains an ambiguous statement and it is unclear whether the feedback component of the intervention was pilot-tested. |
| 10.2 Number of Pages | How long is the feedback report? | Describe. | **Note:** Extract the number of pages for reports. Note whether pages are single-sided or double-sided (if able). If the length is not given (and the form is not available), code as **‘Not Reported’**.  Code as **‘N/A’** if the feedback was provided verbally, involved an e-mail or poster, etc. |
| 10.3 Number of Behaviours | How many behaviours does the feedback address? | 1, 2, 3, 4, 5-9, more than 9, Not Reported, Unclear, N/A, Other (describe as needed) | **Note:** Must be a provider behaviour (a behaviour the provider is expected to change). **'N/A'** applies to non-provider behaviours.  **For the purpose of this question ‘behaviour’ refers to:** -A task, procedure or action completed by the provider to address a specific concern, condition or clinical issue. - If multiple tasks, procedures or actions are considered (i.e. ordering of multiple tests) and the specifics are provided (i.e. the names of the specific tests) this should be coded as multiple behaviours.^5^ - If multiple tasks, procedures or actions are considered (i.e. prescribing of different antibiotics), however the specifics are not provided (i.e. the article focuses generally on prescription of antibiotics) this should be coded as one behaviour (for another example see Herbert et al.^6^). - If a single task, procedure or action is considered (i.e. ordering of benzodiazepines) in multiple contexts (i.e. in combination with other pharmaceuticals, length of the prescription, and whether the benzodiazepine is long-acting),^7^ still code as ONE behaviour. - If the feedback addresses a guideline which involves multiple tasks, procedures or actions, follow the guidance outlined above.  Note: If >9 behaviours are addressed, please specify whether they are of the same general behaviour (i.e. test ordering), or a variety of different behaviours (i.e. different guidelines).  Code as **'Not reported'** if there are no details in the text and the feedback form/additional details are not available.  Code as **‘Unclear’** if the text contains an ambiguous statement and the feedback form/additional details are not available to confirm.  **Examples:**  1) Referral of smoking patients to a quit line^8^ would count as 1 behaviour.  2) Assessing whether physicians ‘ask, ‘advise’, ‘assess’ and ‘assist’ their smoking patients^4^ would count as 4 behaviours.  3) Prescription of antibiotics for pediatric Upper Respiratory Infections, acute bronchitis and purulent rhinitis would count as 3 behaviours. 4) The ordering of Carcino-embryonic antigen, CA-125 and Follicle Stimulating Hormone tests^5^ would count as 3 behaviours. 5) Ordering 2 laboratory tests per clinical issue, for 3 clinical issues would count as 6 behaviours in total (example adapted from Verstappen et al).^3^  **Note:** In some cases a proxy for behaviour may be used. For instance the number of prescriptions filled (or cost of prescriptions)^9^ may be used as a proxy for prescription behaviour. Try to only code provider behaviours. However, if you include a proxy as a ‘behaviour’ ensure that it is clearly associated with provider behaviour. |
| 10.4  Clinical Variables | How many clinical variables were fed back to the recipients? | Describe, Not Reported, Unclear | Includes variables descriptive of the patient (i.e. length of stay, mortality). Does not include behaviours.  Code as **'Not reported'** if there are no details in the text and the feedback form/additional details are not available.  Code as **‘Unclear’** if the text contains an ambiguous statement and the feedback form/additional details are not available to confirm. |
| 10.5 Number of Graphs or Tables | How many graphs or tables are used? | Describe, Not Reported, Unclear | Code as **'Not reported'** if there are no details in the text and the feedback form/additional details are not available. Can also code if only a partial example of a feedback form is given.  Code as **‘Unclear’** if the text contains an ambiguous statement and the feedback form/additional details are not available to confirm. |
| 10.6  Graphical elements | Did the feedback include any graphical elements that lend themselves to misinterpretation? (Ex. Pie charts, 3D graphs, etc.) | Yes, No, Not Reported, Unclear, N/A | Code as **'Yes'** if pie charts, 3D graphs, shadow effects etc. are used.  Code as **'No'** if there are no obvious issues with the presentation of the graphs.  Code as **'Not reported'** if there are no details in the text and the feedback form/additional details are not available. Code as **‘Not Reported’** if the description of the intervention indicates/suggests graphs were used, but no example is provided.  Code as **‘Unclear’** if the text contains an ambiguous statement and the feedback form/additional details are not available to confirm.  Code as **'N/A'** if graphs were not used. |
| **Suggestion #11: Address barriers to feedback use** | | | |
| 11.1  Barriers assessment | Were potential drivers and barriers to recipients engaging with the feedback component of the intervention assessed? | Yes, No, Not Reported, Unclear | Code as **'Yes'** if the authors explicitly state that they completed an assessment of potential drivers and barriers. **Example:** The authors report that they completed an *a priori* focus group with physicians to identify potential barriers to the recipients engaging with the feedback.  Code as **'No'** if there is no indication that the authors completed an assessment of potential drivers and barriers.  Code as **'Not reported'** if there are no details in the text and the feedback form/additional details are not available.  Code as **‘Unclear’** if the text contains an ambiguous statement and the feedback form/additional details are not available to confirm whether the authors completed a drivers/barriers assessment (i.e. the authors discuss barriers identified, but it is unclear whether these barriers were identified before or after feedback was given). |
| 11.2  Barriers assessment- Theory | Was the assessment informed by theory? | Yes, No, Not Reported, Unclear, N/A | Code as **'Yes'** if the authors explicitly mention that the assessment was informed by theory.  Code as **'No'** if the authors indicate that the assessment was not informed by theory (e.g. it was informed by other means).  Code as **'Not reported'** if there are no details in the text and the feedback form/additional details are not available.  Code as **‘Unclear’** if the text contains an ambiguous statement and the feedback form/additional details are not available to confirm whether the assessment was informed by theory.  Code as **'N/A'** if there was no drivers/barriers assessment. |
| 11.3  Engagement assessment | Was there an assessment of whether the recipients engaged with the feedback? | Yes, No, Not Reported, Unclear, Other | Code as **'Yes'** if: • The authors clearly assessed whether the recipients engaged with the feedback. For example, if the recipients were given a questionnaire asking their understanding of the feedback, or if the authors were able to assess how many people clicked on a link to the feedback.  • The authors sent a survey at the same time as the feedback and calculated a response rate.   Code as **'No'** if:  • The authors discuss assessing only a few of the recipient’s engagement with the feedback (i.e. only the team leaders).^10^ • The authors report a response rate from a survey, however the survey was completed at a later time.^8^  Code as **'Not reported'** if:  • The authors do not discuss assessing recipients' engagement with the feedback; there are no details in the text and the feedback form/additional details are not available.  Code as **'Unclear'** if:  • There is an ambiguous statement suggesting the authors assessed recipients' engagement with the feedback, however it is not entirely clear.  • The authors report a response rate from a survey, however it is unclear whether the survey was sent at the same time as the feedback.^8^ |
| 11.4  Engagement assessment- Theory | Was the assessment informed by theory? | Yes, No, Not Reported, Unclear, N/A | Code as **'Yes'** if the authors explicitly mention that the assessment was informed by theory.  Code as **'No'** if the authors indicate that the assessment was not informed by theory, or it is clear that theory was not applied (example: attendance was taken to assess engagement).  Code as **'Not reported'** if there are no details in the text and the feedback form/additional details are not available.  Code as **‘Unclear’** if the text contains an ambiguous statement and the feedback form/additional details are not available to confirm whether the assessment was informed by theory.  Code as **'N/A'** if there was no engagement assessment. |
| **Suggestion #12: Provide short, actionable messages followed by optional detail** | | | |
| 12.1  Actionable messages | Are the summary messages actionable (or described as actionable)? | Yes, No, Not Reported, Unclear, N/A | **Note: The summary message should be a summary of the data.**  **Code as 'Yes' if:**  • The feedback included a summary message(s) which directed the recipient on how to proceed (i.e. the message includes a verb).  **Examples:** 1) “You [need to **increase** your referrals by 9] to achieve the benchmark for this quarter” (adapted from Wadland et al.).^8^  2) Feedback included a manual which “described a number of ways to use the results including discussion with colleagues and assistants...”^11^  3) “A succinct evidence-based message to guide future prescribing.”^6^ 4) “CA125 should not be used to screen, diagnose, or exclude malignancy.”^5^  **Code as 'No' if:**  • The summary message(s) did not direct recipients on how to proceed (i.e. the message did not contain a verb).  **Examples:** 1) “You had 3 referrals for the quarter.”^8^ The benchmark is 9 referrals (adapted from Wadland et al.).^8^  2) The feedback included a letter explaining that "these conditions were being evaluated because little evidence supported antibiotics for their treatment".^12^  **Code as 'Not reported' if:**  • There are no details in the text and the feedback form/additional details are not available.  **Example:** The article describes that the feedback included a cover letter and it is unclear whether actionable summary messages were given.  **Code as 'Unclear' if:**  • The text contains an ambiguous statement and the feedback form/additional details are not available to confirm whether the summary message(s) directed the recipient on how to proceed. |
| 12.2  Additional information | Is there additional, more detailed feedback provided alongside the summary message? | Yes, No, Not Reported, Unclear | Code as **'Yes'** if the feedback component of the intervention provided additional information that was easy to navigate. **Example:** The feedback e-mail contains a hyperlink to additional information. Or, the key message of a feedback report contains the appendix page number where additional information can be found.  Code as **'No'** if the feedback component of the intervention did not provide additional information, or if the additional information is not easy to navigate. **Example:** An intervention which provides 15 pages of additional information on guidelines.  Code as **'Not reported'** if there are no details in the text and the feedback form/additional details are not available.  Code as **‘Unclear’** if the text contains an ambiguous statement and the feedback form/additional details are not available to confirm whether additional information was provided, or if it is unclear whether the additional information was easy to navigate. |
| **Suggestion #13: Address credibility of the information** | | | |
| 13.1 Feedback data source | Does the feedback (or the description of the feedback) indicate who is providing the feedback data? | Yes, No, Not Reported, Unclear | Code as **'Yes'** if:  • The feedback form clearly indicates who is providing the data.  • The article explicitly states that the feedback recipients gave informed consent for data retrieval.  • The data utilized came from the hospital pharmacy or hospital computer system and it is highly likely that the providers were aware this is where the data came from (i.e. they received print outs from the computer system).^2,9^  • Feedback was mailed and the authors state that it showed who was providing the data.  • Feedback was provided verbally or was e-mailed.  Code as **'No'** if:  • The feedback form does not indicate who is providing the data.  Code as **'Not reported'** if: • There is no example of the feedback form available, and the article does not explain who provided the data.  • No example of the feedback form is provided and it is unclear if the feedback form relayed who provided the data.  • The feedback was mailed and it is unclear whether the feedback form relayed who provided the data.  Code as **'Unclear'** if:  • The text contains an ambiguous statement and the feedback form/additional details are not available to confirm.  **Note:** For studies utilizing data from the hospital computer system, you may assume that it was clear to recipients where the data was coming from. |
| 13.2  Comparator source | Does the feedback intervention indicate the source of comparators? | Yes, No, Not Reported, Unclear, Other | Code as **'Yes'** if the feedback form (or the description of the feedback intervention) indicates all sources of comparators. **Example:** The feedback form displays “Benchmark is based on the top 10% of practices referring patients to Quit the Nic and is recalculated for each quarter.”^8^  Code as **'No'** if the source for one or more of the comparators is missing. For example, if the feedback form shows a peer comparison, but there is no definition of who the 'peer' is (i.e. practice, region, all participants in the study).  Code as **'Not reported'** if there are no details in the text and the feedback form/additional details are not available.  Code as **‘Unclear’** if the text contains an ambiguous statement and the feedback form/additional details are not available to confirm whether the source for the comparator(s) was provided. |
| 13.3 Feedback Delivery- Supervisor | Was the feedback intervention delivered by a supervisor or close colleague? | Yes, No, Not Reported, Unclear | Code as **'Yes'** if the feedback intervention was delivered by a supervisor or close colleague. **Example:** An intervention delivered to interns by their respective chief residents.  Code as **'No'** if the feedback intervention was not delivered by a supervisor or close colleague. **Example:** Feedback is delivered by investigators, or by an outside professional organization.  Code as **'Not reported'** if there are no details in the text and the feedback form/additional details are not available.  Code as **‘Unclear’** if the text contains an ambiguous statement and the feedback form/additional details are not available to confirm whether the intervention was delivered by a supervisor or close colleague. |
| 13.4 Feedback Delivery- Organization | Was the feedback intervention supported by a relevant professional organization? | Yes, No, Not Reported, Unclear | Code as **'Yes'** if the feedback intervention was supported by a relevant professional organization (i.e. The feedback form identifies the organization's logo,^8^ or if no example of the feedback form is given, the organization's support is clearly discussed in the article). **Examples:** The Department of Family Medicine - Michigan State University, Blue Cross Blue Shield of Michigan (Quit the Nic Quit Line), etc.^8^ **Note:** Institutional logos used within slideshow presentations were not counted.  Code as **'No'** if no professional organizations are indicated on the feedback form. **Example:** The diagnostic center which provided the data facilitated the social discussions.^3^ **Note:** The organization should be pinned to a recommendation.  Code as **'Not reported'** if there are no details in the text and the feedback form/additional details are not available.  Code as **‘Unclear’** if the text contains an ambiguous statement and the feedback form/additional details are not available to confirm whether the feedback intervention was supported by a professional organization. **Example:** If the intervention included an educational component which clearly involved support from a professional organization (i.e. a brochure produced by the organization), however it is unclear whether they also provided support for the feedback component of the intervention.^12^ |
| **Suggestion #14: Prevent defensive reactions to feedback** | | | |
| 14.1  Defensive reactions | Did the feedback intervention include reassurance that the intervention would not trigger punitive measures? | Yes, No, Not Reported, Unclear, Other | Code as **'Yes'** if the feedback intervention included reassurance that the intervention would not trigger punitive measures. **Example** (Yes): Physicians were assured that this intervention would not involve any punitive measures.  Code as **'Not reported'** if there are no details in the text and the feedback form/additional details are not available.  Code as **‘Unclear’** if the text contains an ambiguous statement and the feedback form/additional details are not available to confirm. |
| **Suggestion #15: Construct feedback through social interaction** | | | |
| 15.1 Social aspect- Development | Did development of the feedback intervention involve members of the target group? | Yes, No, Not Reported, Unclear | Code as **'Yes'** if the development of the feedback component of the intervention involved members of the target group (**Note:** The target group refers to the feedback recipients in the study- i.e. developers and recipients were co-constructing the meaning of the feedback together). **Example:** The authors completed *a priori* focus groups to determine what types of behaviour physicians wanted feedback on.  Code as **'No'** if the development of the feedback component of the intervention did not involve members of the target group.  Code as **'Not reported'** if there are no details in the text and the feedback form/additional details are not available.  Code as **‘Unclear’** if the text contains an ambiguous statement and the feedback form/additional details are not available to confirm whether members of the target group were involved; or for multifaceted interventions, if it is unclear whether target members were involved in the development of the feedback component specifically. |
| 15.2 Social Context | Was the feedback explicitly designed to be received and discussed in a social context? | Yes, No, Not Reported, Unclear, Other | **Code as 'Yes' if:** • There is an explicit statement that the feedback component of the intervention was designed to be received and discussed in a social context. **Note:** This can take place with as few as two people (one-on-one), and may include virtual settings, if two-way communication occurs.  **Code as 'No' if:** • The feedback component of the intervention was clearly not designed to be received and discussed in a social context.  **Code as ‘Not reported’ if:**  • There are no details in the text and the feedback form/additional details are not available.  **Code as ‘Unclear’ if:**  **•** The text contains an ambiguous statement and the feedback form/additional details are not available to confirm whether the feedback component of the intervention was explicitly designed to be received and discussed in a social context.    **Examples** **YES:** The feedback was distributed and discussed during staff meetings, and a statement describing this design is included in the methods section. **NO:** Feedback was mailed to recipients. **UNCLEAR:** The feedback report was mailed but included materials, such as a power-point presentation, to aid in dissemination of the feedback, potentially in a social context.^13^ “Reports were delivered individually to providers 52% of the time, delivered to the group during a provider meeting 30% of the time, and placed in the provider’s mailbox for independent review 18% of the time.”^4^ It was unclear whether the intervention was intended to be delivered in a social context/ with a social discussion. |
| 15.3 How often? | If feedback was provided more than once, how often was feedback received and discussed in a social context? | Every time feedback was provided, Only once, Variably, Not Reported, Unclear, Other (please specify), N/A | Code as **‘N/A’** if feedback was only provided once, or if there is no indication that feedback was received and discussed in a social context. |
| 15.4  Facilitation | If feedback was received and discussed in a social context, was the feedback discussion facilitated by a facilitator? | Yes, No, Not Reported, Unclear, N/A | **Code as 'Yes' if:**  • The feedback intervention involved at least one instance of a facilitated social discussion about the feedback. **Note:** A facilitator may be either internal to the group/institution, or from an external party.  **Code as 'No' if:** • The feedback intervention involved one or more instances of a social discussion (about the feedback), but none of the sessions involved a facilitator.  • The feedback intervention involved another component (i.e. education) involving one or more instances of a facilitated social discussion, however the discussion was not about the feedback.  **Code as 'Not Reported' if:**  • There are no details in the text and the feedback form/additional details are not available.  **Code as 'Unclear' if:** • The text contains an ambiguous statement and the feedback form/additional details are not available to confirm whether a facilitator was present, or it is unclear whether the discussion was about the feedback.  **Code as 'N/A' if:** • There is no indication the feedback was received and discussed in a social context. |
| 15.5 Self-assessment | Did the feedback intervention involve engaging in self-assessment around target behaviours prior to receiving feedback? | Yes, No, Not Reported, Unclear | **Code as 'Yes' if:** • The article discusses a session/survey, which occurred prior to the dissemination of feedback and the authors explicitly state that it involved recipient self-assessment of the target behaviours.  • The intervention involved an educational component prior to the feedback component.  **Code as 'No' if:** • The article discusses a session/survey which involved recipient self-assessment of the target behaviours, however the session/survey took place after the dissemination of feedback.  **Code as 'Not reported' if:**  • There are no details in the text and the feedback form/additional details are not available.  • The article discusses a session/survey, which occurred prior to the dissemination of feedback, however it is not reported whether it involved recipient self-assessment of the target behaviours.  **Code as 'Unclear' if:** • The text contains an ambiguous statement and the feedback form/additional details are not available to confirm.  • The article discusses a session/survey which occurred prior to the dissemination of the feedback, but it is unclear whether the session/survey involved recipient self-assessment of the target behaviours. • The article discusses a session/survey that involved recipient self-assessment of the target behaviours, however it is unclear whether this session/survey took place prior to the dissemination of the feedback.  **Example: YES**: “A pretest was completed by all 50 medical residents prior to distribution of the first newsletter... three questions were asked about individual prescribing charges, five concerning specific prescriptions, and six regarding laboratory testing.”^9^ |
| 15.6 Feedback on Feedback | Did the investigators actively seek feedback from the recipients on the feedback? | Yes, No, Not Reported, Unclear | **Code as ‘Yes’ if:** • The recipients of the feedback intervention were able to provide feedback on the feedback intervention, to the developers (i.e. through a survey, interview, etc.). • The authors of the article discuss a pilot feedback intervention (not necessarily with the feedback recipients from the current study) and explicitly state that the recipients received their own data. • The feedback report requires a response from the recipient, i.e. whether they agree with the feedback.^2^  **Code as ‘No’ if:** • There is no indication that recipients of the feedback intervention were able to provide feedback on the feedback intervention to the developers; and there is no indication that a pilot study was completed using the participants’ own data. • The authors of the article discuss a pilot feedback intervention, however the feedback did not use the recipient’s own data (i.e. a sample form was used).  **Code as 'Not reported' if:**  • There are no details in the text and the feedback form/additional details are not available.  **Code as ‘Unclear’ if:**  • The text contains an ambiguous statement and the feedback form/additional details are not available to confirm. • The authors discuss completing a post-study interview/survey with the feedback recipients, however it is unclear whether they were specifically asked about the feedback form.^4^  • The authors of the article discuss a pilot feedback intervention, however they do not explicitly state whether the recipients received their own data.  **Examples: YES** 1) “Participants who received portraits [feedback] individually completed a brief questionnaire concerning their understanding of the portraits [feedback] and any suggestions for improvement.”^6^ 2) “The physician was required to respond to each item on the report, indicating what action, if any, should be taken. These responses were limited to (1) rescheduling the patient sooner, (2) marking the clinic encounter form... to suggest the physician perform the indicated preventative care on the patient’s next scheduled visit, (3) indicating the protocol was not applicable for this patient (but the physician agrees, in principle, with the protocol), (4) stopping this reminder (the physician disagrees with the protocol), and (5) pulling the patient’s chart for review.”^2^  **UNCLEAR** 1) “Efforts were made to educate providers on the new quitline numbers; however, despite this retraining, **post-study interviews** revealed that many providers were not aware that this service remained available to all clinic patients.”^4^ 2) “Two hundred physicians in 28 PBSGs [Practice-Based Small Groups] joined the trial, with an additional PBSG serving an advisory role to pre-test the materials”;^6^ Not clear whether it was their data or a sample form. |

**References**

1. Brehaut JC, Colquhoun HL, Eva KW, et al. Practice Feedback Interventions: 15 Suggestions for Optimizing Effectiveness. *Ann Intern Med*. 2016;164(6):435-441. doi:10.7326/M15-2248

2. Tierney WM, Hui SL, McDonald CJ. Delayed Feedback of Physician Performance Versus Immediate Reminders to Perform Preventative Care: Effects on Physician Compliance. *Med Care*. 1986;24(8):659-666.

3. Verstappen WHJM, Van Der Weijden T, Dubois WI, et al. Improving Test Ordering in Primary Care: The Added Value of a Small-Group Quality Improvement Strategy Compared With Classic Feedback Only. *Ann Fam Med*. 2004;2(6):569-575. doi:10.1370/afm.244

4. Bentz CJ, Bayley KB, Bonin KE, et al. Provider feedback to improve 5A’s tobacco cessation in primary care: A cluster randomized clinical trial. *Nicotine Tob Res*. 2007;9(3):341-349. doi:10.1080/14622200701188828

5. Thomas RE, Croal BL, Ramsay C, Eccles M, Grimshaw J. Effect of enhanced feedback and brief educational reminder messages on laboratory test requesting in primary care: a cluster randomised trial. *Lancet*. 2006;367(9527):1990-1996. doi:10.1016/S0140-6736(06)68888-0

6. Herbert CP, Wright JM, Maclure M, et al. Better Prescribing Project: a randomized controlled trial of the impact of case-based educational modules and personal prescribing feedback on prescribing for hypertension in primary care. *Fam Pract*. 2004;21(5):575-581. doi:10.1093/fampra/cmh515

7. Pimlott NJG, Hux JE, Wilson LM, Kahan M, Li C, Rosser WW. Educating Physicians to Reduce Benzodiazepine Use by Elderly Patients: A Randomized Controlled Trial. *CMAJ*. 2003;168(7):835-839.

8. Wadland WC, Holtrop JS, Weismantel D, Pathak PK, Fadel H, Powell J. Practice-Based Referrals to a Tobacco Cessation Quit Line: Assessing the Impact of Comparative Feedback vs General Reminders. *Ann Fam Med*. 2007;5(2):135-142. doi:10.1370/afm.650

9. Hershey CO, Goldberg HI, Cohen DI. The Effect of Computerized Feedback Coupled with a Newsletter upon Outpatient Prescribing Charges: A Randomized Controlled Trial. *Med Care*. 1988;26(1):88-94.

10. Cohen DI, Jones P, Littenberg B, Neuhauser D. Does Cost Information Availability Reduce Physician Test Usage?: A Randomized Clinical Trial with Unexpected Findings. *Med Care*. 1982;20(3):286-292. doi:10.2307/3764297

11. Vingerhoets E, Wensing M, Grol R. Feedback of patients’ evaluations of general practice care: a randomised trial. *Qual Heal Care*. 2001;10:224-228. doi:10.1136/qhc.0100224..

12. Mainous AG, Hueston WJ, Love MM, Evans ME, Finger R. An Evaluation of Statewide Strategies to Reduce Antibiotic Overuse. *Fam Med*. 2000;32(1):22-29.

13. Beck CA, Richard H, Tu J V, Pilote L. Administrative Data Feedback for Effective Cardiac Treatment: AFFECT, A Cluster Randomized Trial. *JAMA*. 2005;294(3):309-317. doi:10.1001/jama.294.3.309

1. An ‘Other’ option was added as required. [↑](#footnote-ref-1)
