# Supplementary material for: How well do critical care audit and feedback interventions adhere to best practice? Development and application of the REFLECT-52 evaluation tool
Source: Implement Sci. 2021 Aug 17;16:81. doi: 10.1186/s13012-021-01145-9 (PMC8369748; doi:10.1186/s13012-021-01145-9)
Supplement: Supplementary file 4 — Additional File 4. PRISMA flow diagram of the study selection. [file 13012_2021_1145_MOESM4_ESM.docx]

Additional File 4: PRISMA Flow Diagram^1^ outlining the selection of articles (reprinted from Implementation Science^2^ [Open Access])

**
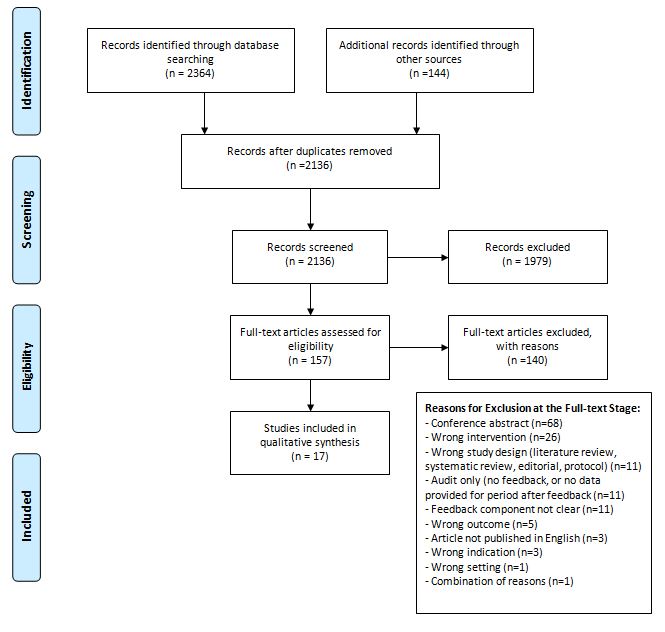
**

**References**

1. Moher D, Liberati A, Tetzlaff J, Altman D, The PRISMA Group. Preferred Reporting Items for Systematic Reviews and Meta-Analyses: The PRISMA Statement. *PLoS Med*. 2009;6(7):e1000097. doi:10.1371/journal.pmed1000097.
2. Foster M, Presseau J, McCleary N, Carroll K, McIntyre L, Hutton B, et al. Audit and feedback to improve laboratory test and transfusion ordering in critical care: a systematic review. Implement Sci. 2020;15(1):46.
