# Supplementary material for: How well do critical care audit and feedback interventions adhere to best practice? Development and application of the REFLECT-52 evaluation tool
Source: Implement Sci. 2021 Aug 17;16:81. doi: 10.1186/s13012-021-01145-9 (PMC8369748; doi:10.1186/s13012-021-01145-9)
Supplement: Supplementary file 5 — Additional File 5. Assessment of remaining descriptive items. [file 13012_2021_1145_MOESM5_ESM.docx]

**Additional File 5. Assessment of Remaining Descriptive Items**

**Table 1. ‘Nature of the Desired Action’ descriptive criteria items**

| **Criteria item** | **Frequency (out of 17 interventions)** |
| --- | --- |
| ***Level at which the priority was set*** |  |
| Institutional | 4 |
| Departmental | 2 |
| Unit | 3 |
| National | 1 |
| Healthcare system | 1 |
| Unclear | 2 |
| ***Average age of the feedback data*** |  |
| Hours | 2 |
| Days | 2 |
| Weeks | 3 |
| Months | 2 |
| Days and weeks | 3 |
| Weeks, months and unclear | 1 |
| Unclear | 4 |
| ***Time interval between receipt of feedback reports (if provided more than once)*** |  |
| Hours | 1 |
| Days | 2 |
| Days and weeks | 1 |
| Weeks | 3 |
| Weeks and variable | 2 |
| Weeks, months and variable | 1 |
| Unclear/ variable | 3 |
| Not applicable | 4 |

**Table 2. ‘Nature of the Data Available’ descriptive criteria items**

| **Criteria item** | **Frequency (out of 17 interventions)** |
| --- | --- |
| ***Level of the group*** |  |
| Unit | 5 |
| Team (multiple units) | 2 |
| Ward and institution | 1 |
| Unit and department | 1 |
| Institution (ICUs) and potentially provider groups | 1 |
| Not reported | 2 |
| Not applicable | 5 |
| ***Number of comparators*** |  |
| One | 1 |
| Two | 4 |
| Unclear, but at least one | 2 |
| Unclear, but at least two | 2 |
| Not applicable | 8 |
| ***Types of comparator (*frequency out of 15 comparators)*** |  |
| Own group’s previous performance | 7* |
| Group’s performance | 1* |
| Other’s performance | 3* |
| Target | 2* |
| Other (own group’s average over time) | 1* |
| Other (peers’ individual performance) | 1* |
| Not applicable | 8 |

ICUs= intensive care units

**Table 3. ‘Feedback Display’ descriptive criteria items**

| **Criteria item** | **Frequency (out of 17 interventions)** |
| --- | --- |
| ***Number of clinical variables fed back*** |  |
| None | 3 |
| At least one | 1 |
| At least four | 2 |
| Five | 1 |
| Not reported | 10 |
| ***Number of graphs or tables used*** |  |
| None | 1 |
| One graph | 1 |
| Two graphs | 1 |
| Two graphs, one table | 1 |
| Three graphs, one table | 1 |
| Unclear, at least one graph | 1 |
| Unclear, at least two graphs | 1 |
| Not reported | 10 |
| ***Length of the feedback report*** |  |
| One page | 1 |
| Two reports, one page each | 1 |
| Not reported | 9 |
| Not applicable (e.g. email, presentation, poster) | 6 |
| ***Number of behaviours addressed by the feedback*** |  |
| At least one behaviour | 7 |
| Two behaviours | 1 |
| At least two behaviours | 2 |
| At least three behaviours | 1 |
| Three to five behaviours | 2 |
| Five to nine behaviours | 3 |
| At least seven behaviours, up to 44 | 1 |

**Table 4. ‘Delivering the Feedback’ descriptive criteria items**

| **Criteria item** | **Frequency (out of 17 interventions)** |
| --- | --- |
| ***If provided more than once, how often was feedback received and discussed in a social context*** |  |
| Every time | 4 |
| Variably (aimed for every time for one of two types of feedback) | 1 |
| Unclear | 1 |
| Not applicable | 11 |
